# Thecal and Intracellular Ultrastructure of *Prorocentrum majusculum* n. sp. (Prorocentrales, Dinophyceae), a New Spiny *Prorocentrum* from Northwest Pacific

**DOI:** 10.1111/jeu.70141

**Published:** 2026-11-14

**Authors:** Koyo Kuwata, Urban Tillmann, Chiho Funaki, Takako Masuda, Kazutaka Takahashi, Mitsunori Iwataki

**Affiliations:** ^1^ Graduate School of Agricultural and Life Sciences University of Tokyo Tokyo Japan; ^2^ Alfred Wegener Institute Helmholtz Center for Polar and Marine Research Bremerhaven Germany; ^3^ Japan Fisheries Research and Education Agency Shiogama Miyagi Japan

**Keywords:** mucocyst, mucus trap, peduncle, periflagellar area, pyrenoid, trichocyst

## Abstract

Small, spiny *Prorocentrum* species of the *P. cordatum* group, comprising *P. cordatum* , *P. insidiosum*, *P. pervagatum*, *P. shikokuense*, *P. spinulentum*, and *P. thermophilum* , have recently been described or reinvestigated, and some of them are mixotrophic and produce globular mucus traps. Here, a mucus trap‐forming *Prorocentrum* from the Northwest Pacific was characterized by LM, SEM, and TEM. Cells were laterally compressed, round, and 17–29 μm in length. Cells had a nucleus in the posterior part and brownish chloroplasts with two pyrenoids. Thecal plates were ornamented with small spines and two types of pores. The periflagellar area consisted of eight platelets, most bearing one wing‐like protrusion, two on platelets 1 and 8, and absent on platelet 4. TEM observations showed mucocysts containing fibrous material, trichocysts of different sizes, and the peduncular apparatus. Pyrenoids were surrounded or penetrated by thylakoids. Granules discharged from the small pores and trichocysts from the large pores were observed. In rDNA phylogeny, this species fell within the *P. cordatum* group, yet formed a distinct clade separated from described species. It can be distinguished from other members of the *P. cordatum* group by larger cell size and periflagellar structure; therefore, we propose it as *P. majusculum* n. sp.

**Abbreviations**
ap: accessory pore (= auxiliary or apical pore)
BI: Bayesian inference
DIC: differential interference contrast
fp: flagellar pore
ITS: internal transcribed spacer
LM: light microscopy
LMR: longitudinal microtubular root
LSU: large subunit
ML: maximum likelihood
MSP: microtubular strand of peduncle
PSC: peduncle striated collar
SC: striated collar
SEM: scanning electron microscopy
SSU: small subunit
TEM: transmission electron microscopy
TSR: transverse striated root
UFBoot: ultrafast bootstrap

## 1 Introduction

Morphological differentiation of small marine planktonic *Prorocentrum* Ehrenberg species, representing mostly round cells with a spiny or tuberculate thecal surface, is challenging. These dinoflagellate species are difficult to distinguish solely by using light microscopy (LM). Important diagnostic features, as currently defined, include the presence and type of apical projections, details of the thecal plate surface structure, the arrangement of thecal pores, and the structure of the periflagellar area, and in most cases require scanning electron microscopy (SEM). Therefore, many small *Prorocentrum* species remain inadequately described, relying on early descriptions and lacking detailed morphological or genetic characterization. This has resulted in persistent taxonomic uncertainty, exemplified by *P. balticum* (Lohmann) Loeblich, a species that is often reported in the literature, but whose type is not well described, underscoring the need for epitypification (Tillmann, Gottschling, et al. 2023; Tillmann, Wietkamp, et al. 2023; Mordret et al. 2025). Nevertheless, several small *Prorocentrum* species— *P. thermophilum* F. Gómez, Tangcheng Li, Hu. Zhang & Senjie Lin, *P. pervagatum* Tillmann, Hoppenrath & Gottschling, *P. spinulentum* Tillmann, Gottschling & Hoppenrath, and *P. insidiosum* Tillmann, Larsson & Hallegraeff—have been recently described based on a combination of LM, SEM, and molecular methods (Gómez et al. 2023; Tillmann, Gottschling, et al. 2023; Tillmann, Wietkamp, et al. 2023; Larsson et al. 2025). All these species are members of a well‐supported monophyletic clade which also includes the ubiquitous and bloom‐forming harmful *P. cordatum* (Ostenfeld) J.D.Dodge (= *P. minimum* (Pavillard) J.Schiller) and is thus known as the “*cordatum* group” (Gómez et al. 2023; Tillmann, Gottschling, et al. 2023; Tillmann, Wietkamp, et al. 2023; Larsson et al. 2025). Current molecular phylogenies of the “*cordatum* group” indicate the presence of additional yet undescribed or misidentified species (Gómez et al. 2023; Tillmann, Gottschling, et al. 2023; Tillmann, Wietkamp, et al. 2023; Funaki et al. 2024; Larsson et al. 2025).

Some species of the *P. cordatum* group have attracted particular attention because of their mixotrophic capabilities. Dinoflagellates frequently exhibit mixotrophy, combining phototrophy with phagotrophy, and display a remarkable diversity of feeding strategies, including direct engulfment, tube feeding, and pallium feeding (e.g., Hansen and Tillmann 2020). Although two species, *P. micans* Ehrenberg and *P. cordatum* , have long been known as photosynthetic phagotrophs (Stoecker et al. 1997; Shim et al. 2011; Johnson 2015), recent studies have revealed that certain members of the *P. cordatum* group—*P. insidiosum* (as *P*. cf. *balticum*), *P. pervagatum*, and *P. cordatum* —employ a highly specialized and sophisticated feeding strategy. They produce large, globular mucus traps (mucospheres) to capture prey (Larsson et al. 2022, 2025; Tillmann, Mitra, et al. 2023). These structures enhance prey encounter rates and facilitate ingestion through a peduncle (Larsson et al. 2022; Tillmann, Mitra, et al. 2023), a tubular appendage used to feed on immobilized prey. This foraging strategy is particularly advantageous in low‐turbulence, oligotrophic environments where traditional prey capture is limited (Larsson et al. 2022, 2025; Tillmann, Mitra, et al. 2023). Moreover, the production and subsequent sinking of prey‐laden mucospheres by *Prorocentrum* make a measurable contribution to the vertical flux of organic carbon, influencing the biological carbon pump (Larsson et al. 2022, 2025), making this prey capture and feeding mode an ecologically important feature.

In 2022, new strains of *Prorocentrum* were isolated from the Kuroshio‐Oyashio transition zone of the Northwest Pacific, and the formation of globular mucus traps was observed (Funaki et al. 2024). These strains were phylogenetically close to Australian strains of what was provisionally named *P*. cf. *balticum* (Larsson et al. 2022), and the Japanese strains were thus provisionally designated as *P*. cf. *balticum* as well (Funaki et al. 2024). In this study, we characterize this *Prorocentrum* species through detailed morphological observations using LM, SEM, and TEM, and through phylogenetic analyses based on SSU, ITS, and LSU rDNA sequences.

## 2 Materials and Methods

### 2.1 Culture and Light Microscopy (LM)

Detailed information on sample collection, cell isolation, and strain maintenance is described in Funaki et al. (2024). Two strains of *Prorocentrum*, KWT167 and KWT168, were isolated from the Pacific Ocean off northeastern Japan (39°30.040′ N, 146°30.010′ E) in July 2022. The clonal cultures were maintained in half‐strength Daigo IMK medium (Wako, Tokyo, Japan), with a salinity of 30 at 20°C and light intensity of 40–50 μmol photons m^−2^ s^−1^ under a 12:12 h light:dark cycle regime.

Cells were observed using a Zeiss Axioskop 2 light microscope (Carl Zeiss, Jena, Germany) equipped with a digital camera Axiocam MRc5 (Carl Zeiss) or with an Axiocam 305 color (Carl Zeiss). Live cells were observed by differential interference contrast (DIC), and thecal plates were examined using epifluorescence microscopy after cells had been stained with solophenylflavine (Direct Yellow 96) (Chomérat et al. 2017). Cell length and depth in dense but healthy, growing strains during the exponential phase were measured using ImageJ (Schneider et al. 2012). The shape and location of the nucleus were determined after formalin‐fixed cells were stained with 4′,6‐diamidino‐2‐phenylindole (DAPI, 0.1 μg mL^−1^ final concentration) for 10 min.

### 2.2 Scanning Electron Microscopy (SEM)

The protocols followed those described by Tillmann, Wietkamp, et al. (2023). Cells were collected by centrifugation of 50 mL culture (5810R; Eppendorf) at 3220 × ***g*** for 10 min. The supernatant was removed, and the cell pellet was resuspended in 60% ethanol prepared with seawater (final salinity ca. 13) in a 2 mL microtube at 4°C for 1 h to strip off the outer cell membrane. Cells were further collected by centrifugation (5415R, Eppendorf) at 16,000 × ***g*** for 5 min and resuspended and fixed in a 60:40 mixture of deionized water and seawater (final salinity ca. 13) with the addition of formaldehyde (1% final concentration) and stored at 4°C for 3 h. Cells were collected on polycarbonate filters (Millipore Merck; Darmstadt, Germany; 25 mm, 3 μm pore‐size) in a filter funnel, on which all subsequent washing and dehydration steps were performed. A total of eight washing steps (2 mL MilliQ‐deionized water each) were followed by a dehydration series in ethanol (30%, 50%, 70%, 80%, 95%, and 100%) for 10 min each. Filters were dried with hexamethyldisilazane (HMDS), first in 1:1 HMDS: EtOH, followed by twice 100% HMDS, and then stored in a desiccator under gentle vacuum. Finally, filters were mounted on stubs, sputter‐coated (Emscope SC500; Ashford, UK) with gold–palladium, and viewed at 10 kV under an SEM (FEI Quanta FEG 200; Eindhoven, The Netherlands).

For morphometric measurements, flat areas of 4 μm × 4 μm were randomly selected to quantify the areal density of spines. The number of large and small pores was counted from half of the internal surface area of thecal plates exposed in internal view. All visible regions of the theca were used to randomly measure spine length and pore size. The procedure used for measuring the diameters of large and small pores from SEM images is illustrated in Figure S1.

### 2.3 Transmission Electron Microscopy (TEM)

For TEM preparation, cells of strain KWT168 were fixed, dehydrated, and embedded according to Kuwata, Lum, et al. (2024). Cells were prefixed for 30 min in 2% (v/v) glutaraldehyde in 0.2 M Na‐cacodylate buffer (pH 7.5, final concentration) at 4°C. After gentle centrifugation, the pellet was rinsed with decreasing sucrose concentrations of 0.3 M, 0.15 M, and pure buffer (10–20 min each). It was then postfixed overnight at 4°C with 1% (w/v) OsO_4_ in 0.1 M Na‐cacodylate buffer. Fixed cells were dehydrated in an ethanol series (30%, 50%, 75%, 90%, and 95%) for 10–15 min each, and twice in 100% ethanol for 30 min each. Cells were embedded in a low‐viscosity resin (EM Japan, Tokyo, Japan) via propylene oxide and were subsequently polymerized at 70°C for 12 h. Sections were cut using an Ultracut S UTC ultramicrotome (Leica Microsystems, Wetzlar, Germany) and mounted on a Formvar‐coated grid. After staining with uranyl acetate and lead citrate, the sections were examined using a JEM‐1400 TEM (JEOL, Tokyo, Japan) at an acceleration voltage of 120 kV, and images were captured using a CCD camera (EM‐14650DR173, JEOL, Tokyo, Japan).

### 2.4 Molecular Phylogeny

For ribosomal DNA (rDNA) sequencing, isolated cells were directly transferred into PCR tubes after rinsing with distilled water (Kuwata et al. 2025). For the amplification and sequencing of the ITS region, SSU rDNA, and LSU rDNA (D1–D3), primers reported in previous studies were used (Kawami et al. 2006; Iwataki et al. 2007; Takahashi et al. 2015). Extracted DNA was amplified in a reaction containing 0.05 μL of ExTaq polymerase (Takara, Shiga, Japan), according to the manufacturer's protocol. The thermal cycling conditions included an initial denaturation at 95°C (3 min), followed by 35 cycles of the following three steps: 95°C (1 min), 48°C (1 min), and 72°C (3 min), and a final elongation step of 72°C (6 min), on a VeritiPro thermal cycler (Thermo Fisher). Amplicons were purified using a QIAquick PCR Purification Kit (Qiagen Genomics, Bothell, WA, USA), following the manufacturer's protocol. Amplicons were sequenced by Eurofins Genomics Inc. (Tokyo, Japan). SSU rDNA, ITS region, and LSU rDNA sequences were aligned with sequences from GenBank using a default setting in MAFFT v.7 and were then manually corrected. Phylogenetic trees were inferred by Bayesian inference (BI) using MrBayes v3.2.7a (Ronquist et al. 2012) under the GTR + I + G model. Two independent runs of four Markov chains were run for 2,000,000 generations (concatenated dataset) or 1,000,000 generations (single‐marker datasets), sampling every 100 generations, and the first 2500 sampled trees were discarded as burn‐in. Convergence was confirmed when the average standard deviation of split frequencies fell below 0.01. Maximum likelihood (ML) analyses were performed using IQ‐TREE v2.3.6 (Minh et al. 2020). For the concatenated dataset, a partitioned model (SSU, ITS, LSU) was applied, and the best‐fit models selected by ModelFinder (Kalyaanamoorthy et al. 2017) under BIC were TN + F + I + R2 (SSU), TIM2 + F + G4 (ITS), and TN + F + R4 (LSU). For the single‐marker datasets, the selected models were TN + F + R2 (SSU), TIM2 + F + G4 (ITS), and TIM2 + F + R4 (LSU). Node support was assessed with 1000 ultrafast bootstrap replicates (UFBoot; Hoang et al. 2018). The outgroups selected were *Azadinium poporum* Tillmann & Elbrächter (LC881721) and *Scrippsiella acuminata* (Ehrenberg) Kretschmann, Elbrächter, Zinssmeister, Soehner, Kirsch, Kusber & Gottschling (HM483396). The pairwise genetic distance in the ITS region was calculated using MEGA 11 with a total of 554 positions (Stecher et al. 2020; Tamura et al. 2021). ITS2 secondary structure of *P. majusculum* strain KWT167 and *P. insidiosum* strain UTSPH3C1 was predicted using RNAfold from the ViennaRNA Package version 2.6.4 with default parameters to predict the minimum free energy (MFE) structures under the temperature condition of 25°C.

## 3 Results

Two strains (KWT167 and KWT168) were examined using LM and SEM, and strain KWT168 was further examined using TEM. No morphological differences other than cell size were observed between the strains. Strain KWT167 was selected as the basis for the type material and is illustrated in detail herein. Morphometric measurements were conducted primarily on this strain, unless otherwise stated.

*Prorocentrum majusculum* Kuwata, Iwataki & Tillmann n. sp. Figures 1, 2, 3, 4.

**FIGURE 1:**
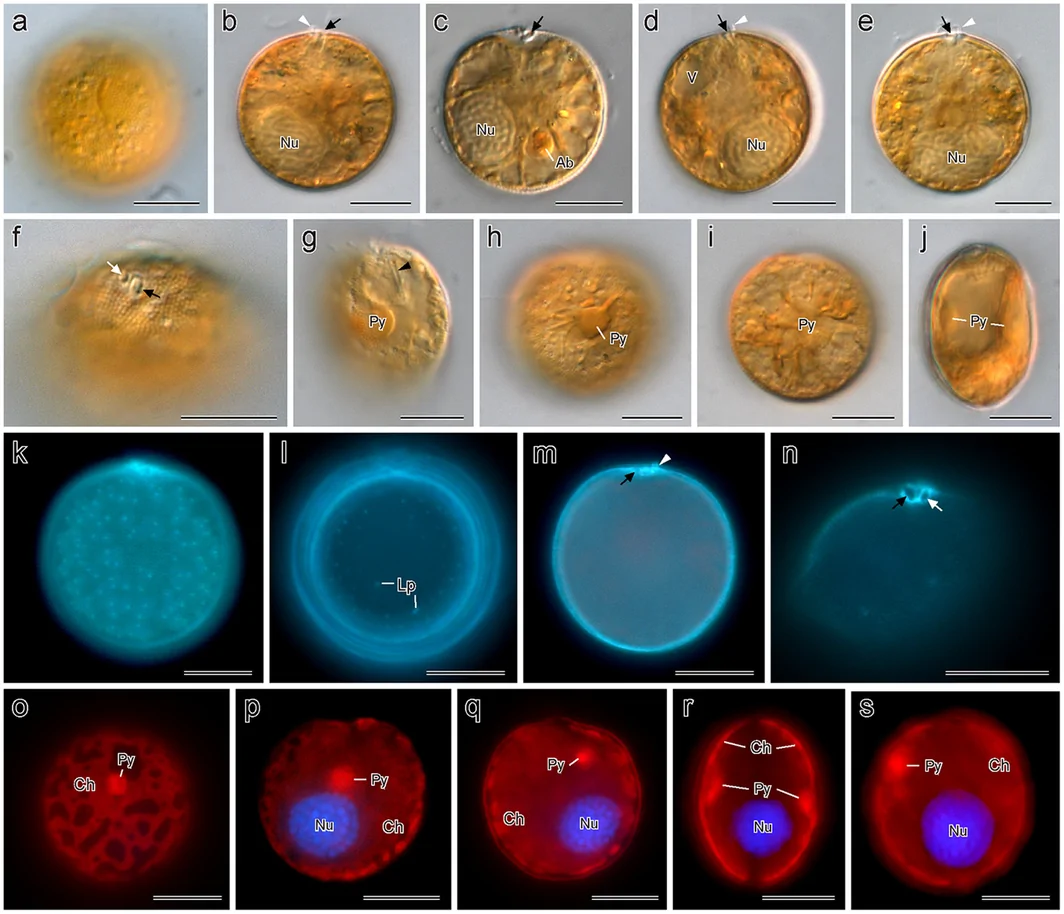
Light and fluorescence microscopy of *Prorocentrum majusculum* n. sp., strain KWT167. (a–j) Light microscopy of surface view (a), right side views (b, c), left side views (d, e), apical view (f), and middle focal planes (g–j), showing accessory pore (white arrows), flagellar pore (black arrows), trichocyst (black arrowhead), and wing‐like protrusion of platelet 1 (white arrowheads). (k–n) Fluorescence microscopy of thecae stained with solophenylflavine, showing surface with thecal pores (k, l) and periflagellar area (m, n). (o–s) Fluorescence microscopy of cells stained with DAPI. Ab, Accumulation body; Ch, chloroplast; Lp, large pore; Nu, nucleus; Py, pyrenoid; V, vacuole. Scale bars = 10 μm.

**FIGURE 2:**
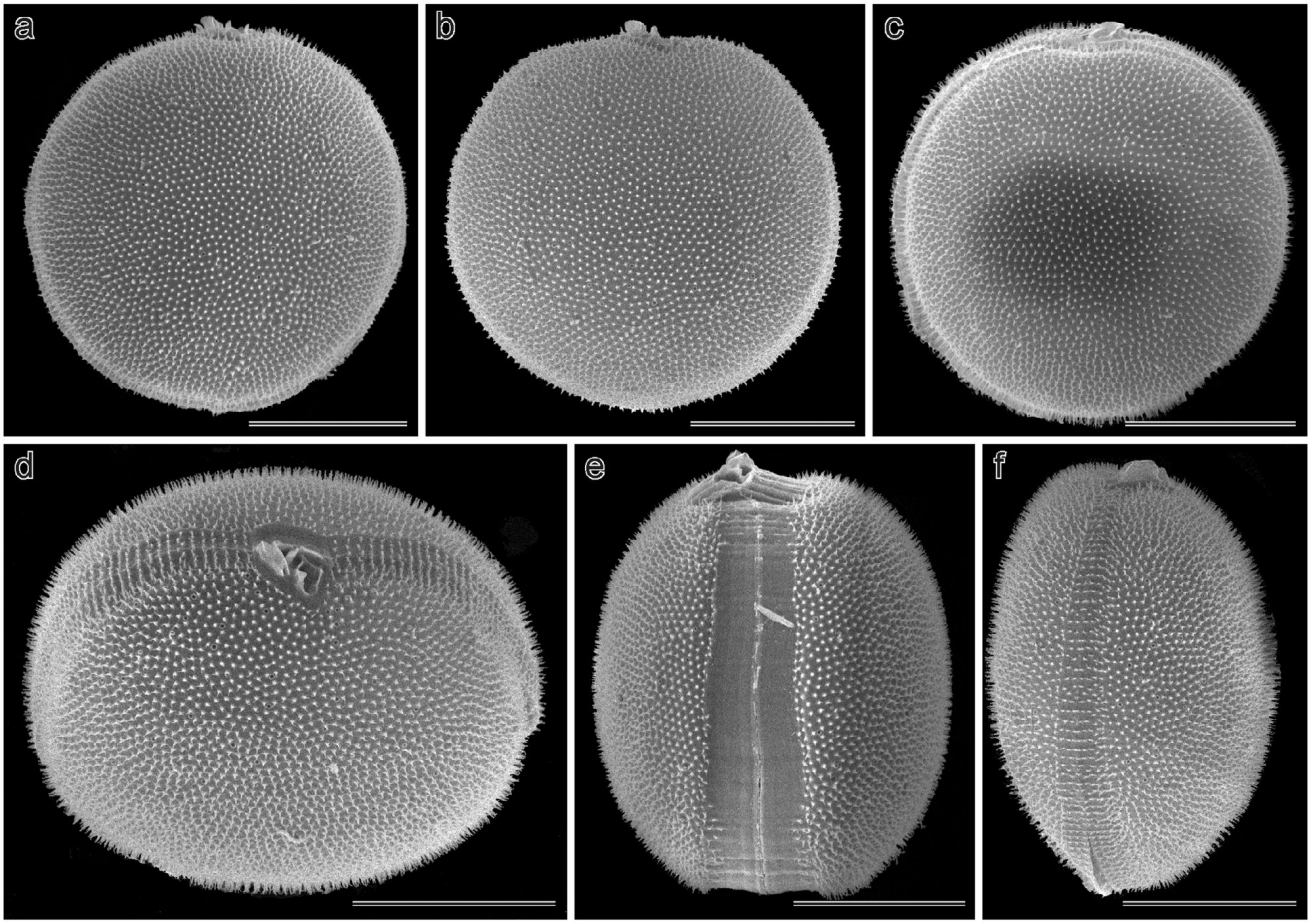
Scanning electron microscopy of *Prorocentrum majusculum* n. sp., strain KWT167. (a, b) Right‐lateral views. (c) Left lateral view. (d) Apical right‐lateral view. (e) Ventral view. (f) Dorsal view. Scale bars = 10 μm.

**FIGURE 3:**
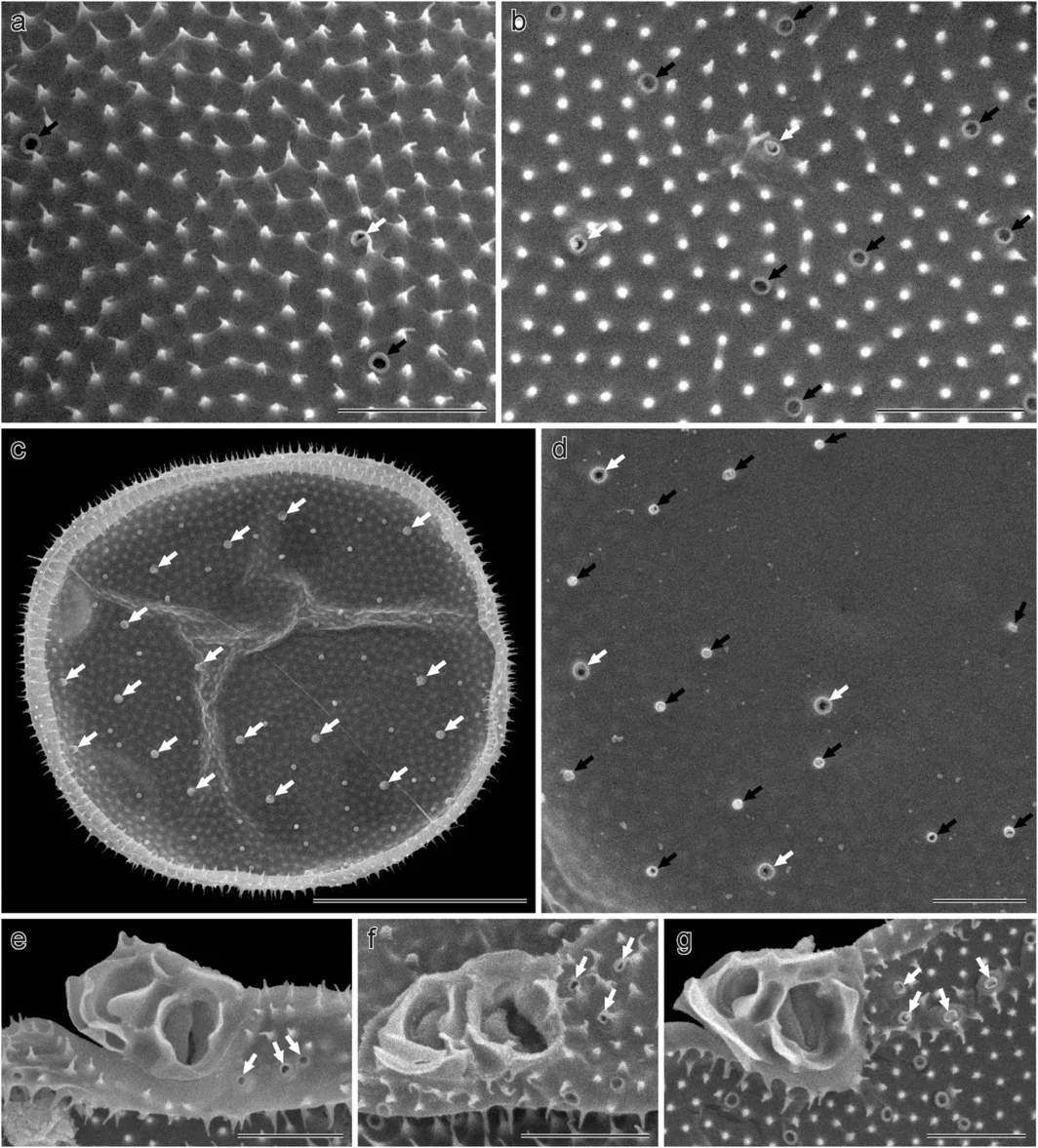
Scanning electron microscopy of *Prorocentrum majusculum* n. sp., strain KWT167. (a, b) Cell surfaces showing spines, small thecal pores (black arrows), and large thecal pores (white arrows). (c) Internal view of the thecal plate. (d) Detailed view of the lining of thecal pores. (e–g) Groups of the large thecal pores near the periflagellar area. Scale bars = 2 μm (a, b, d–g), 10 μm (c).

**FIGURE 4:**
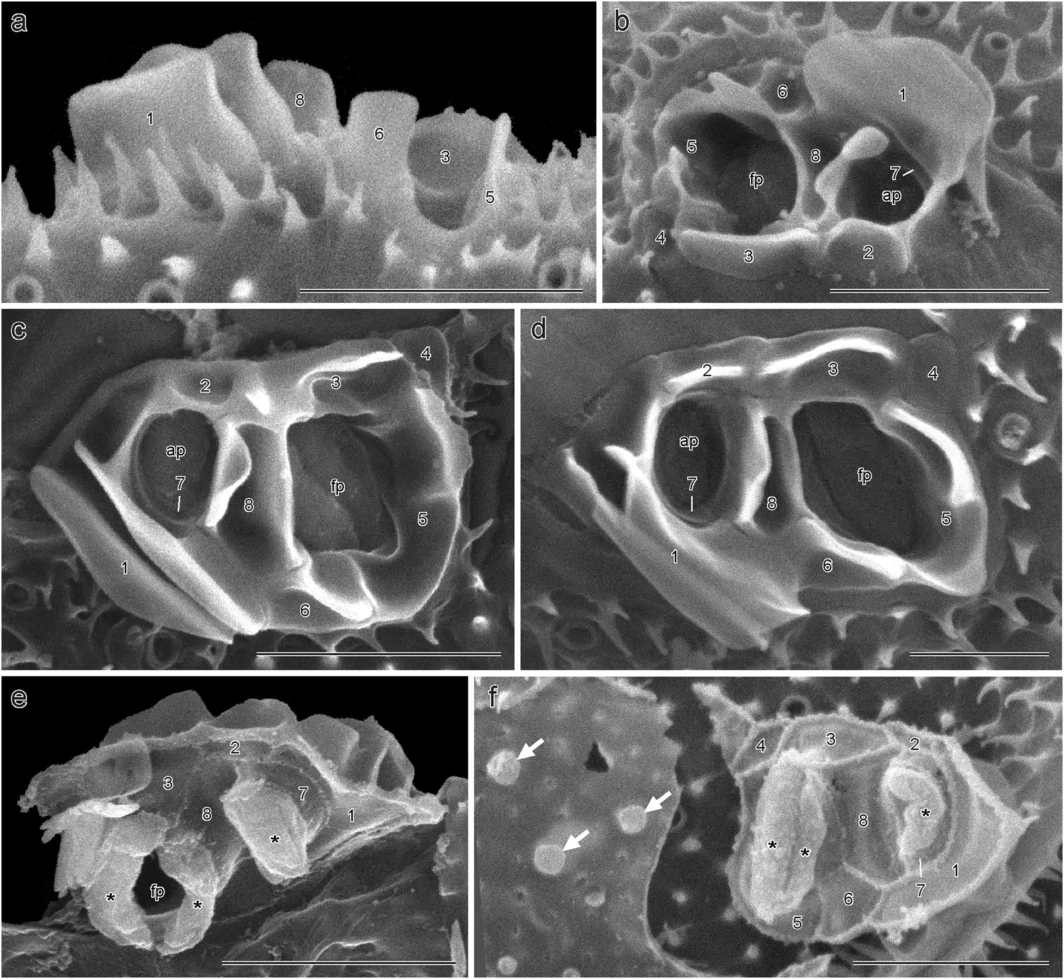
Periflagellar area of *Prorocentrum majusculum* n. sp., strains KWT167 (a–c, f) and KWT168 (d, e). (a) Right lateral view. (b) Left lateral view. (c, d) Detailed apical views. (e, f) Internal views. Numbers (1–8) denote the periflagellar platelets, and other labels are accessory pore (ap), flagellar pore (fp), large thecal pores (white arrows), and lip‐like structure (asterisks). Scale bars = 2 μm.

Description: Small, thecate dinoflagellate. Cells round in lateral view, disc‐shaped in dorsal/ventral view, and 17–29 μm in length and depth. Nucleus posterior, variably positioned from central to dorsal. Two orange‐brown chloroplasts, each with one centrally located round pyrenoid. Mucocysts containing fibrous material are abundant. Trichocysts are variable in size. Thecal plate surface ornamented with short spines and two types of pores. Periflagellar area composed of eight platelets, a small, oval accessory pore on the dorsal side, and a large, elongated flagellar pore on the ventral side. Two parallel, wing‐like protrusions on platelets 1 and 8. A single wing‐like protrusion on each of the other platelets except platelet 4.

Holotype: SEM stub prepared from clonal strain KWT167 (designated CEDiT2026H218) deposited at the Senckenberg Research Institute and Natural History Museum, Centre of Excellence for Dinophyte Taxonomy, Germany.

Reference material: Formalin‐fixed sample prepared from clonal strain KWT167 (designated CEDiT2026RM219), deposited at the Senckenberg Research Institute and Natural History Museum, Centre of Excellence for Dinophyte Taxonomy, Germany.

Additional material: Live cultures of strains KWT167 and KWT168 have been deposited at the National Institute for Environmental Studies (NIES) as NIES‐4707 (KWT167) and NIES‐4708 (KWT168).

Type locality: Pacific Ocean off northeastern Japan (39°30.040′ N, 146°30.010′ E).

Etymology: The epithet (Lat. majusculum = somewhat large, larger than usual) refers to the noticeably enlarged disc‐shaped cell relative to its close congeners.

DNA sequence: LC779630 (SSU rDNA), LC779631 (ITS and LSU rDNA) (strain KWT167), LC958961 (SSU, ITS, and LSU rDNA) (strain KWT168).

### 3.1 Light Microscopy (LM)

Cells were round in lateral view, and occasionally the apical part of the cell was slightly flat (Figures 1 and S2). The cell surface was spiny (Figure 1a); thecal pores were not visible in LM but became apparent under epifluorescence in solophenylflavine‐stained cells (Figure 1k,l). Cell size of strain KWT167 ranged 23.8–28.8 μm (mean 25.7 ± 1.1 μm, *n* = 50) in length and 23.3–28.8 μm (mean 25.4 ± 1.2 μm, *n* = 50) in depth. Cell size of strain KWT168 ranged 17.3–20.0 μm (mean 18.5 ± 0.8 μm, *n* = 30) in length and 16.9–19.9 μm (mean 18.2 ± 0.8 μm, *n* = 30) in depth. The oval to elongated nucleus was located posteriorly, either on the dorsal side (*n* = 17; Figure 1b–d,p,q) or centrally within the posterior half of the cell (*n* = 15; Figure 1e). The flagellar pore (Figure 1b–f,m,n) and a wing‐like protrusion on platelet 1 (Figure 1b,d,e,m) were visible. From the apical view, the accessory pore was also visible (Figure 1f,n). A spherical accumulation body was occasionally observed (Figure 1c). A vacuole was also sometimes visible, mainly in the anterior ventral region (Figure 1d). Many rod‐like trichocysts were observed beneath the thecal plate, and sometimes they were ejected during observation (Figures 1g and S2b,e). One large, round, and orange pyrenoid was located beneath each thecal plate, from which yellow to brownish, reticulate chloroplasts radiated peripherally (Figure 1g–j,o–s).

### 3.2 Scanning Electron Microscopy (SEM)

The cell surface was covered with small spines (Figures 2, 3, 4). Spine length ranged from 0.30 to 0.76 μm (mean 0.51 ± 0.07 μm, *n* = 22 thecae, 428 spines in total), with spine density ranging from 3.6 to 5.0 μm^−2^ (mean 4.3 ± 0.3 μm^−2^, *n* = 20 thecae, 100 areas in total) (Table 1). The spines on the plate surface were conical, occasionally bearing a short filiform extension at the apex (Figure 3a). In addition, spines were connected at their bases by small ridges, variably developed and sometimes barely visible (Figure 3a,b). The periflagellar area, as a distinct structure, was mainly positioned towards the right thecal plate (Figure 2d). Several wing‐like protrusions on the periflagellar area were prominent (Figure 2). The intercalary band lining the suture of both lateral plates was variable in width (Figure 2d–f). Usually, transverse striations consisting of 4–8 latitudinal rows of spines, which were shorter than those on thecal plates, were observed (Figure 2d,f), but in some cells, no such spines were observed on their band (Figure 2e).

**TABLE 1:** *Prorocentrum majusculum* n. sp., strain KWT167, morphometry of thecal projections and pores.

	Spine length (μm)	Spine density (μm^−2^)	Small pore size (μm)		Large pore size (μm)		Large/small pore ratio	Estimated thecal pore number per theca	
			External	Internal	External	Internal		Small pore	Large pore
Mean ± SD	0.51 ± 0.07	4.3 ± 0.3	0.16 ± 0.01	0.21 ± 0.01	0.40 ± 0.02	0.37 ± 0.02	0.21 ± 0.05	86 ± 19.5	19 ± 5.7
Min. to max.	0.30–0.76	3.6–5.0	0.11–0.26	0.13–0.32	0.35–0.47	0.26–0.47	0.13–0.32	68–130	12–28
Number of measured	22 thecae, 428 spines in total	20 thecae, 100 areas in total	25 thecae, 311 pores in total	22 thecae, 1119 pores in total	25 thecae, 47 pores in total	23 thecae, 384 pores in total	21 thecae, 52–129 pores per theca	15 thecae	15 thecae

Two types of thecal pores were recognized on the plate surface, designated as small and large pores in the sense of Larsson et al. (2025) (Figure 3 and Table 1). Small pores with a crateriform rim ranged from 0.11 to 0.26 μm (mean 0.16 ± 0.01 μm, *n* = 25 thecae, 311 pores in total) in diameter and 68 to 130 pores per plate (mean 86 ± 19.5 plate^−1^, *n* = 15 thecae). Pores were distributed almost randomly, with slightly lower densities in the center of the plate and higher densities at the periphery (Figure 3c). Large pores had a distinct ring structure (diameter: 0.35–0.47 μm; mean 0.40 ± 0.02 μm, *n* = 25 thecae, 47 pores in total) with a small central opening delimited by a crateriform rim. There were 12 to 28 such large pores per plate (mean 19 ± 5.7 plate^−1^, *n* = 15 thecae; Figure 3a). In the internal view, the two types of pores were clearly observed and distinguished as rounded protuberances (Figure 3c). Large pores were usually open, whereas small pores exhibited both open and closed apertures (Figure 3d). In the internal view, the protuberances of the small pores measured 0.13–0.32 μm in diameter (0.21 ± 0.01 μm, *n* = 22 thecae, 1119 pores in total), whereas those of the large pores measured 0.26–0.47 μm (0.37 ± 0.02 μm, *n* = 23 thecae, 384 pores in total). Large pores often formed groups of two to five (most commonly three) in the anterior ventral region near the periflagellar area on the right thecal plate (Figures 3e–g and 4f).

The periflagellar area was composed of eight platelets, a small, oval accessory pore (ap), and a large, elongated flagellar pore (fp) (Figure 4). Platelet 1 was the largest, and platelet 4 was the smallest. Platelet 7 was generally difficult to observe from external views because it was very narrow and deeply positioned, forming the dorsal lower lining of the ap (Figure 4b–d). From internal views, it was often observed (Figure 4e,f). The ap was surrounded by platelets 1, 2, 7, and 8, and the fp was surrounded by platelets 3, 5, 6, and 8. There were one or two wing‐like protrusions on each platelet except platelet 4. On platelet 1, two long wings ran in parallel and in straight lines on the right side (Figure 4a–d). Platelet 8 also possessed two protrusions along the side towards the ap and fp (Figure 4c,d). The protrusions on platelet 1 were usually the highest (Figure 4a,b), but in some cases were similar in height to those of other platelets. In the internal view, the ap and fp canals appeared as two inwardly extending ridges directed downward and connected to a lip‐like structure (Figure 4e,f). Both open and closed lip‐like structures were observed (Figure 4e,f).

Schematic illustrations of the cell in right lateral view and details of the periflagellar area are shown in Figure 5.

**FIGURE 5:**
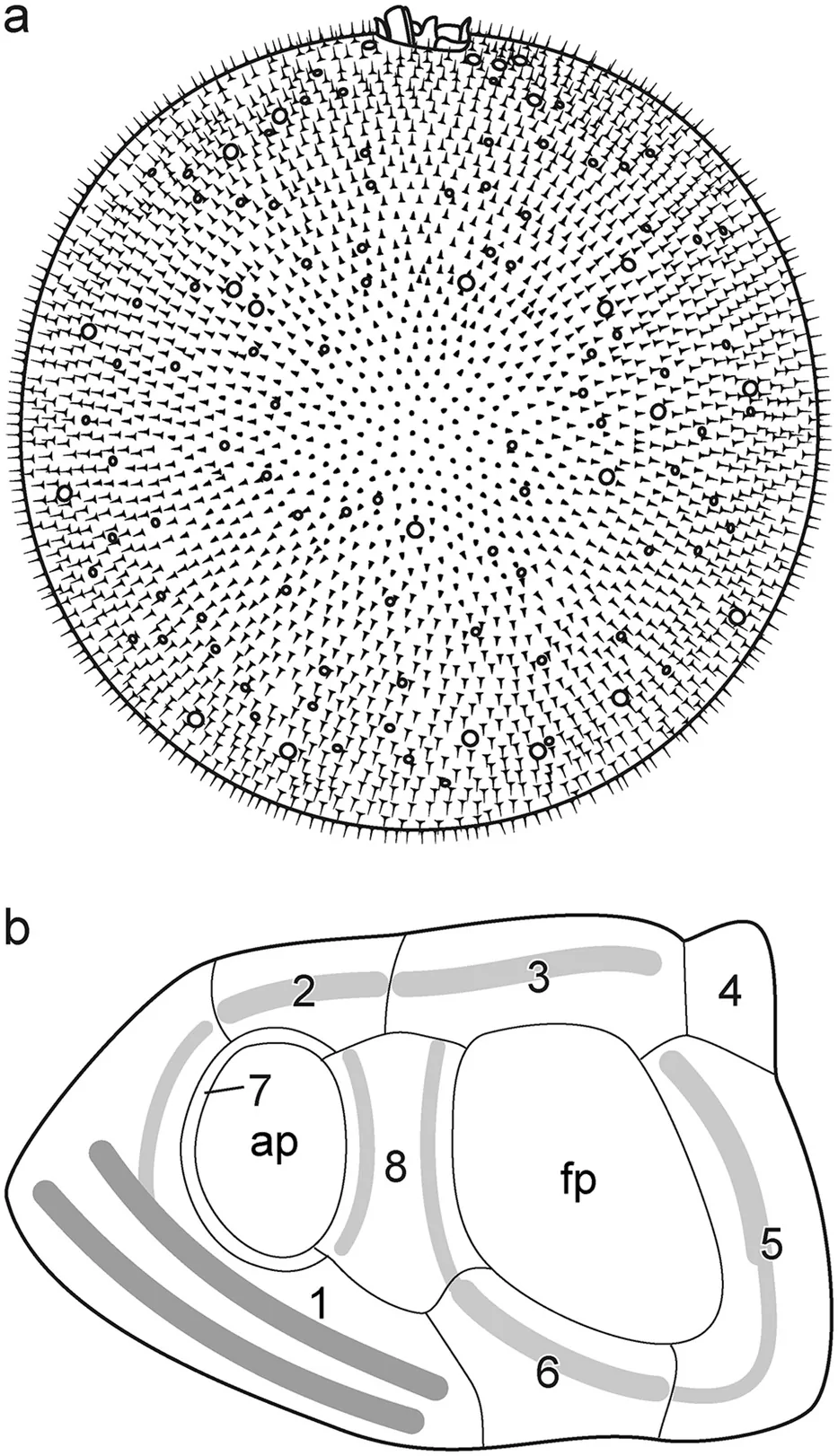
Schematic illustrations of *Prorocentrum majusculum* n. sp. (a) Right side view. (b) Detail of the periflagellar area. Dark and light gray lines indicate taller and shorter wing‐like protrusions, respectively.

### 3.3 Transmission Electron Microscopy (TEM)

TEM observations revealed typical dinoflagellate organelles, including a dinokaryotic nucleus, mitochondria with tubular cristae, and chloroplasts with two or three appressed thylakoids (Figure 6). Chloroplasts were distributed along the cell periphery (Figure 6a). Bulged pyrenoids were laterally located near the center of each thecal plate and were surrounded by a cup‐shaped starch sheath (Figure 6a–c). They were usually surrounded by several single thylakoids (Figure 6a,b) and occasionally penetrated by two‐appressed thylakoids arrayed at even intervals (Figure 6c). Numerous Golgi apparatuses were clustered above the nucleus (Figure 6a). Trichocysts were numerous and variable in size and radially arranged around the cell periphery (Figure 6d). The side length of quadrilateral trichocysts was 318–425 nm (mean 378 ± 28 nm, *n* = 55). Vesicles containing trichocyst‐like structures associated with fibrous material were also observed (Figure S3a,b); these structures were presumably trichocyst precursors. Fibrous vesicles were also observed; these were elongated and contained fibrous material arranged parallel to the longitudinal axis of the vesicle (Figure S3a). Mucocysts were abundant in the cytoplasm and contained densely packed bunches of fibrillar material radially arrayed (Figures 6a,e,f and S3c,d). Electron density of the fibrillar material was variable; some appeared opaque and others more lucent (Figure S3c,d).

**FIGURE 6:**
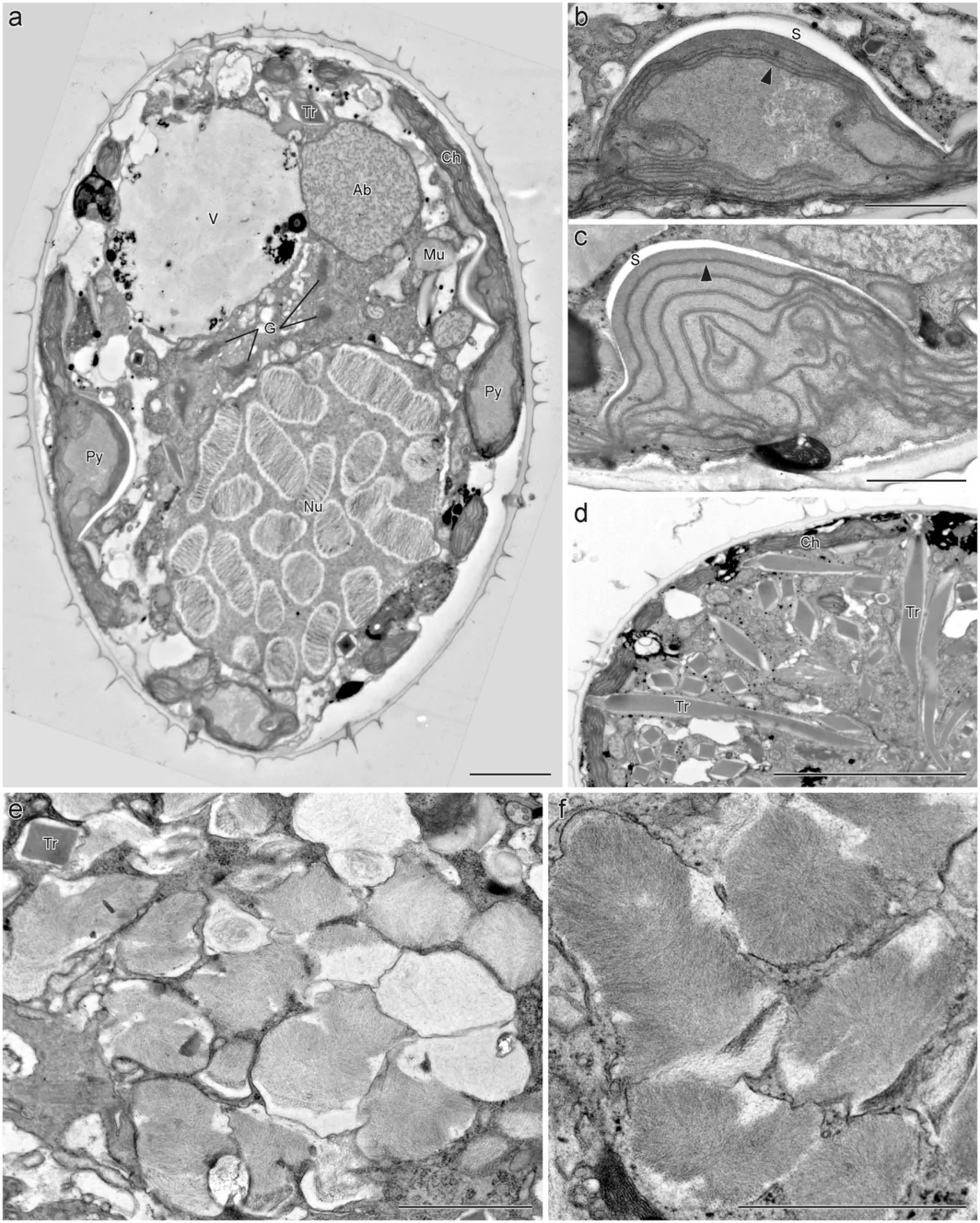
Transmission electron microscopy of *Prorocentrum majusculum* n. sp., strain KWT168. (a) Longitudinal section from dorso‐ventral view showing accumulation body (Ab), chloroplast (Ch), Golgi apparatus (G), mucocyst (Mu), nucleus (Nu), pyrenoid (Py), and vacuole (V). (b, c) Pyrenoid with surrounding (b) and penetrated (c) thylakoids (arrowhead) and starch sheath (S). (d) Trichocysts (Tr). (e) Mucocysts. (f) Fibrous components of mucocysts. Scale bars = 5 μm (a, d), 1 μm (b, c, e, f).

Ultrastructural features of both small and large thecal pores were observed (Figure 7a–d). Small pores were associated with an underlying chamber containing electron‐dense granular material, which was released through the pore (Figure 7a), although pores lacking such a chamber were also observed (Figure 7b). A slightly swollen and electron‐lucent structure was present beneath the small pore at its basal region (Figure 7a,b). Marginal protrusion of external openings was ca. 50 nm (Figure 7a,b). In large pores, discharging trichocysts approximately 135 nm in width were observed (Figure 7c). The trichocyst showed a striated structure with intervals of approximately 15 nm, and every fourth striation was more electron‐dense (Figure 7c). Empty large pores were also observed (Figure 7d). Marginal protrusion of external openings was ca. 300 nm (Figure 7c,d).

**FIGURE 7:**
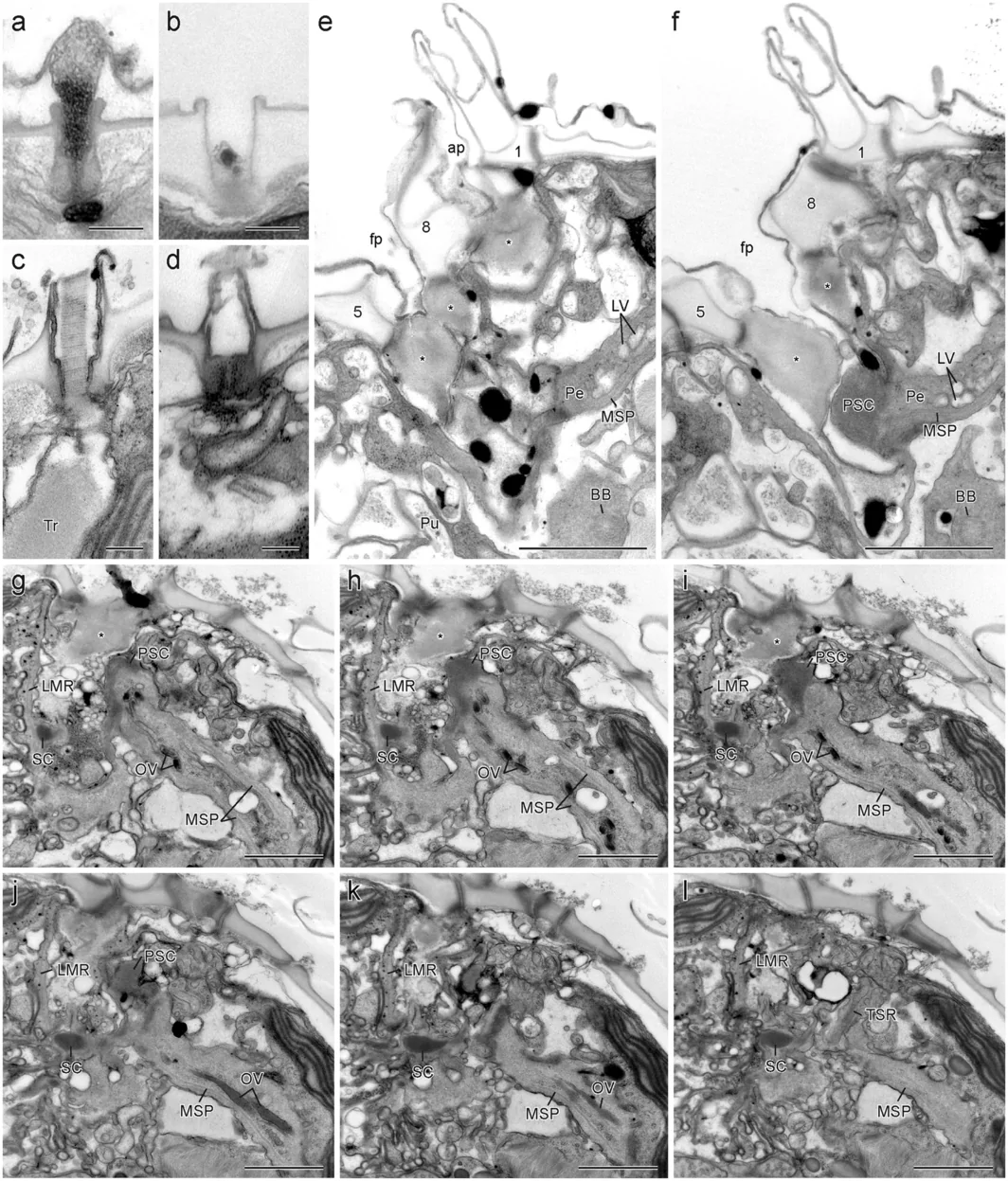
Transmission electron microscopy of *Prorocentrum majusculum* n. sp., strain KWT168. (a–d) Longitudinal sections of small (a, b), large pores (c, d), and empty pores (b, d). (e, f) Periflagellar area with the lip‐like structure (asterisks) and periflagellar platelets (1, 5, 8) forming flagellar and accessory canals. (g–l) Serial sections of flagellar apparatus and peduncle. Abbreviations: Accessory pore (ap), basal body (BB), flagellar pore (fp), electron‐lucent vesicle (LV), longitudinal microtubular root (LMR), microtubular strand of peduncle (MSP), electron‐opaque vesicle (OV), peduncle (Pe), peduncle striated collar (PSC), pusule (Pu), striated collar (SC), trichocyst (Tr), and transverse striated root (TSR). Scale bars = 200 nm (a–d), 1 μm (e–l).

The lip‐like structures surrounding the flagellar and accessory canals were electron‐opaque (Figure 7e,f), and pusules were observed near the flagellar canal (Figure 7e). A peduncle containing microtubules, corresponding to the microtubular strand of the peduncle (MSP), was observed (Figure 7e–l). The peduncle was approximately 680 nm in diameter and was connected to the flagellar canal (Figure 7e,f). A prominent peduncle striated collar (PSC) was present at its distal end (Figure 7f–j). The peduncle contained either electron‐lucent vesicles (labeled LV in Figure 7e,f) or electron‐opaque vesicles (labeled OV in Figure 7g–k). Portions of the flagellar apparatus were also observed (Figure 7e–l). Basal bodies (BB), together with a longitudinal microtubular root (LMR), a transverse striated root (TSR), and a striated collar (SC), were identified (Figure 7e–l).

### 3.4 Molecular Phylogeny

In Bayesian inference (BI) phylogeny of SSU‐ITS‐LSU rDNA concatenated sequences (Figure 8), two *P. majusculum* sequences formed a robust clade (BI/ML = 1.00/100) in the *P. cordatum* group (1.00/100), which consisted of *P. cordatum* (0.91/99), *P. insidiosum* (1.00/100), *P. pervagatum* (0.74/99), *P. shikokuense* (0.98/96), *P. spinulentum*, *P. thermophilum* (1.00/100), and *P. majusculum* (Figure 8). Within this group, the clade of *P. majusculum* was most closely related to that of *P. insidiosum* from Southeast Australia (1.00/98). *Prorocentrum majusculum* and *P. insidiosum* were then related to *P. thermophilum* (1.00/91), followed by *P. pervagatum* (1.00/100).

**FIGURE 8:**
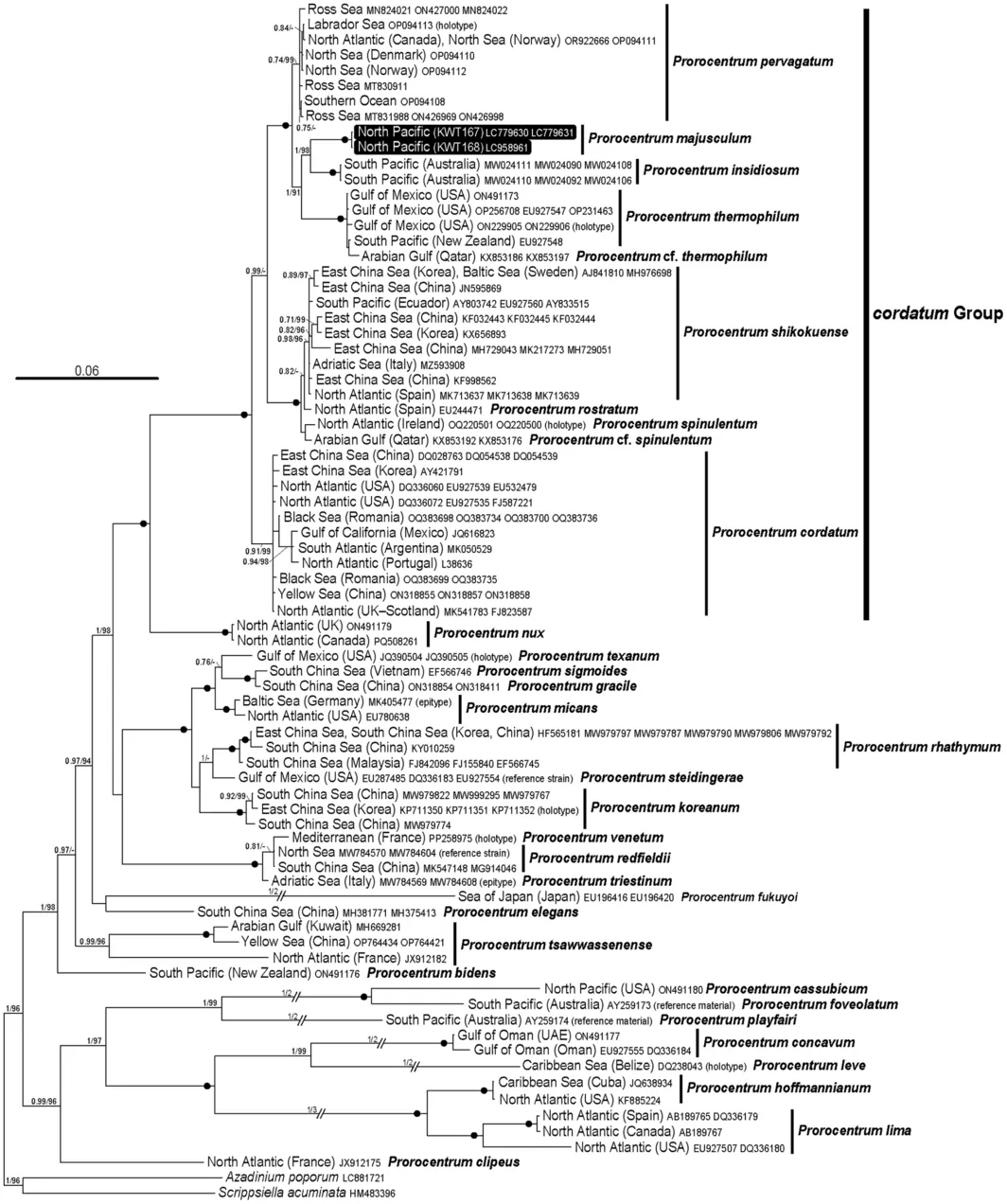
Bayesian inference (BI) tree of *Prorocentrum* species based on concatenated SSU‐ITS‐LSU rDNA sequence (4000 bp). Posterior probabilities (> 0.70) of BI and ultrafast bootstrap support values (≥ 90%) of maximum likelihood (ML) are given at nodes (BI/ML). Black circles indicate maximum support (BI/ML = 1.00/100). DNA sequences determined in the present study are highlighted in black.

Affinity of the *P. cordatum* group was supported in the phylogenies of SSU rDNA (1.00/100), ITS region (1.00/100), and LSU rDNA (1.00/99), and their topologies resembled the concatenated tree (Figures S4–S6). However, in the SSU rDNA tree (Figure S4), the phylogenetic placement of the two *P. majusculum* sequences was not supported, and their relationship to *P. insidiosum* or *P. thermophilum* remained unresolved. In the ITS tree (Figure S5), *P. majusculum* was related to *P. insidiosum* (1.00/96) and then *P. pervagatum* (0.75/−), but *P. thermophilum* was not related to these species in the *P. cordatum* group. In the LSU rDNA tree (Figure S6), the related species of *P. majusculum* were *P. insidiosum* (0.77/91), then *P. pervagatum* (unsupported), and *P. thermophilum* (1.00/100).

SSU rDNA sequences of *P. majusculum* could be distinguished from all other *Prorocentrum* species. However, in the partial SSU rDNA sequences, *P. majusculum* could not be distinguished from some *Prorocentrum* species. In the V4 region (419 bp), *P. majusculum* shared an identical sequence with *P. cordatum* , *P. koreanum* M.‐S.Han, S.Y.Cho & P.Wang, *P. steidingerae* F.Gómez, D.Qiu & S.Lin, *P. micans* , *P. rhathymum* A.R.Loeblich III, Sherley & R.J.Schmidt, and *P. thermophilum* . In the V9 region (169 bp), *P. majusculum* shared an identical sequence with *P. cordatum* , *P. insidiosum*, *P. pervagatum*, *P. shikokuense*, *P. spinulentum*, and *P. thermophilum* .

Genetic distances estimated from pairwise *p*‐distance between *P. majusculum* and the other six species in the *P. cordatum* group ranged from 0.040 (*P. insidiosum*) to 0.126 (*P. spinulentum*) (Table S1). ITS2 secondary structure of the two species, *P. majusculum* and *P. insidiosum*, showed four main helices (I–IV) with three to five unpaired bases at each apex (Figure S7). There were two hemi‐compensatory base changes (hCBC; a substitution on one side of a base pair that maintains pairing), one each in helices I and III. No CBC (compensatory base change) was found.

## 4 Discussion

### 4.1 Morphology of *Prorocentrum majusculum* n. sp.

Recent physiological studies on two sets of planktonic *Prorocentrum* strains, both provisionally identified as *P*. cf. *balticum* without accompanying detailed morphological analyses, independently reported the formation of mucus traps for mixotrophic prey capture (Larsson et al. 2022; Funaki et al. 2024). While the organism investigated in the first study (Larsson et al. 2022) has since been formally described as *P. insidiosum* (Larsson et al. 2025), we here assign a new scientific name, based on detailed morphological investigations, to the second provisional *P*. cf. *balticum* taxon reported by Funaki et al. (2024). As already indicated by phylogenetic analysis (Funaki et al. 2024), the two taxa formerly referred to as *P*. cf. *balticum* are closely related. However, our detailed morphological analyses reveal clear and consistent differences which justify the description of *P. majusculum* as a new species distinct from the Australian taxon *P. insidiosum*.

A recurring challenge in *Prorocentrum* taxonomy is the differentiation of new species from historically described taxa that lack ultrastructural and molecular data. With its nearly perfectly round shape in lateral view, *P. majusculum* differs from distinctly oval species (e.g., *P. aporum* (Schiller) Dodge; or *P. bidens* Schiller), elongated species (e.g., *P. asymmetricum* (Wislouch) Krakhmalny; *P. oblongum* (Schiller) Taylor), or species with a pointed antapex (e.g., *P. scutellum* Schröder) (Schiller 1918, 1928; Wislouch 1924). Other species with a round lateral outline differ markedly in size; *P. balticum* is significantly smaller (9–12 μm in length; Lohmann 1908), whereas others, such as *P. caspicum* Henckel (60–110 μm in length; Henckel 1909), *P. magnum* (Gaarder) Dodge (72–92 μm in length; Gaarder 1954), or *P. obtusum* Ostenfeld (ca. 44 μm in length; Ostenfeld 1908), are considerably larger than *P. majusculum*. *Prorocentrum lenticulatum* (Matzenauer) Taylor (36 μm in length) and *P. texanum* Henrichs, Steidinger, P.S.Scott & L.Campbell (25–38 μm in length, for culture strains) are only slightly larger, but both differ from *P. majusculum* by having a foveate rather than spiny thecal surface (Matzenauer 1933; Osorio‐Tafall 1942; Taylor 1976; Henrichs et al. 2013). Additional species with a round lateral outline include *P. sphaeroideum* Schiller (36–40 μm diameter; Schiller 1928) and *P. robustum* Osorio‐Tafall (32–39 μm in length; Osorio‐Tafall 1942). Dodge's (1982) synonymization of both taxa with *P. scutellum* is questionable, as *P. scutellum* has an asymmetric and slightly pointed antapex. Nevertheless, both *P. sphaeroideum* and *P. robustum* differ from *P. majusculum* not only by their slightly larger size but also by distinct morphological details: *P. sphaeroideum* bears a single, prominent spine and has plates with only a few pores (Schiller 1928), whereas *P. robustum* exhibits coarse, circular depressions scattered over the entire surface (Osorio‐Tafall 1942). Finally, *P. majusculum* closely resembles *P. rotundatum* Schiller in size and overall outline (Schiller 1918). Schiller (1918) reported dimensions of 16–24 μm in length and 16–21 μm in width, resulting in a size overlap with *P. majusculum*, particularly with the smaller strain KWT168 (17.3–20.0 μm). *Prorocentrum rotundatum* has been reported from multiple regions (Adriatic Sea, Mediterranean, and Mozambique Channel; Dodge 1975); however, these records generally lack detailed morphological documentation. According to the original description (Schiller 1918), *P. rotundatum* possesses a large, lobed chloroplast and a posteriorly located nucleus, features also observed in *P. majusculum*. However, *P. rotundatum* is characterized by a very prominent spine bearing a sail‐like structure (resembling *P. micans* ), and an adjacent knob‐like protuberance (Schiller 1918), clearly distinguishing it from *P. majusculum*.

Based on the molecular phylogeny, *P. majusculum* is a member of the *P. cordatum* group. To date, six species with detailed morphological and molecular characterization are recognized within this group: *P. cordatum* , *P. insidiosum*, *P. pervagatum*, *P. shikokuense*, *P. spinulentum*, and *P. thermophilum* (Pertola et al. 2003; Shin et al. 2019; Gómez et al. 2023; Tillmann, Gottschling, et al. 2023; Tillmann, Wietkamp, et al. 2023; Larsson et al. 2025). All species in this group possess spiny or knob‐like ornamentation and pores on the thecal plates, distinguishing them from other phylogenetic lineages within *Prorocentrum*, such as the *P. nux* lineage ( *P. nux* Puigserver & Zingone and *P. pseudonux* F.Gómez, Graff van Creveld, Francoeur, S.N.Coesel, M.J.Schatz, Armbrust & Senjie Lin) or the *P. triestinum* group, which both have smooth thecal plates, and the *P. micans* group with a foveate plate surface (Tillmann et al. 2019, 2022; Mordret et al. 2025; Gómez et al. 2026). The spiny thecal plate surface of *P. majusculum*, together with other morphological details as outlined below, is fully consistent with this morphological framework and thus clearly supports its placement within the *P. cordatum* group.

Within the *P. cordatum* group, a characteristic feature of *P. majusculum* is its relatively large cell size (17.3–28.8 μm in length and 16.9–28.8 μm in depth), which is particularly striking compared with its closest relative, *P. insidiosum* (Figure 9). However, the two strains of *P. majusculum* differed in cell size, with a mean length of 25.7 μm (KWT167) and 18.5 μm (KWT168), and the size range of KWT168 overlaps with the upper range of *P. cordatum* (Table 2). *Prorocentrum shikokuense* has a cell length (15.3–19.9 μm) overlapping with that of the smaller strain KWT168 (17.3–20.0 μm) (Roselli et al. 2019; Shin et al. 2019); however, its elongated, lanceolate shape clearly differs from the round lateral outline of *P. majusculum*. In addition to cell size, *P. majusculum* can be differentiated from other species of the *P. cordatum* group by the length of thecal spines, the number of pores, and details of the periflagellar area.

**FIGURE 9:**
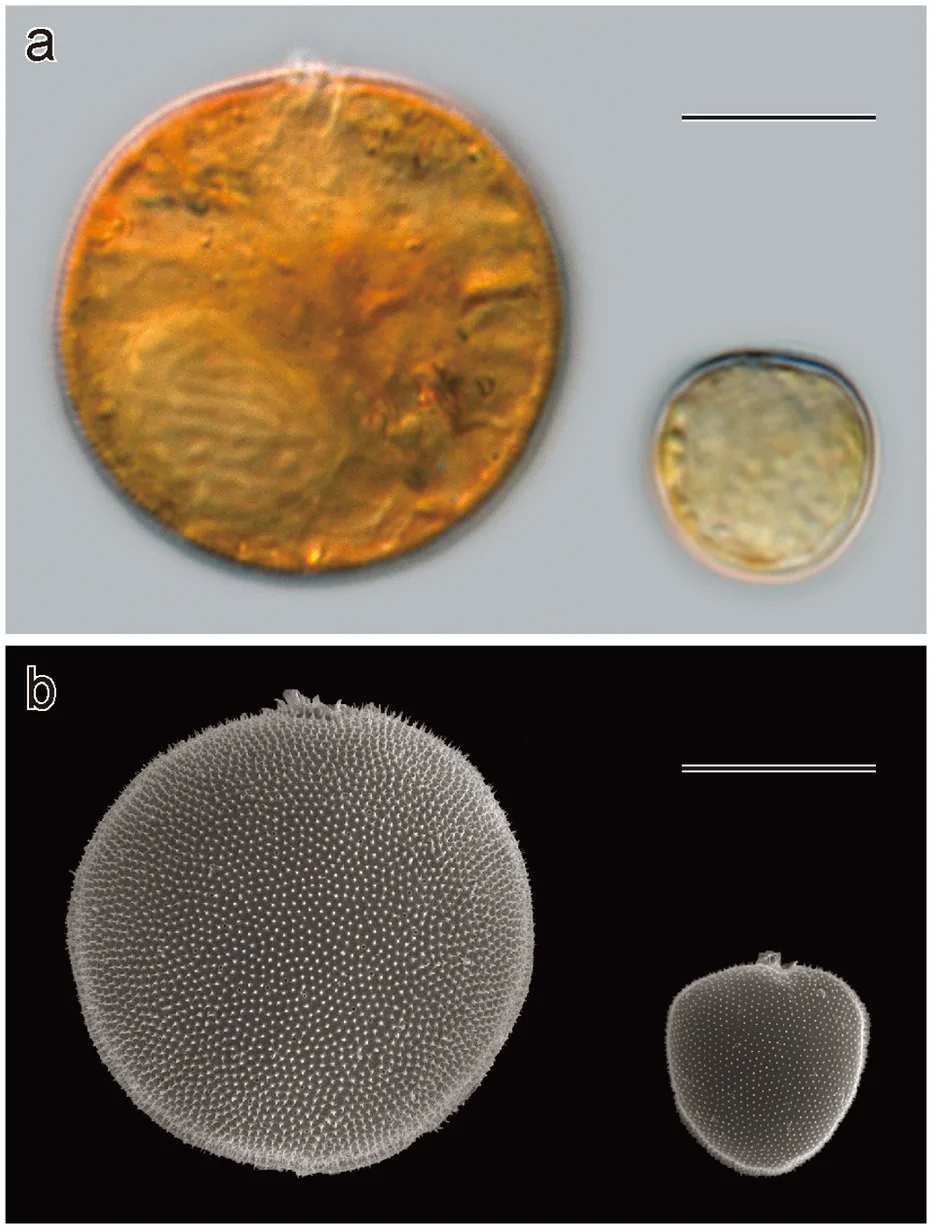
Morphological and size comparison of *Prorocentrum majusculum* n. sp. (left) and *P. insidiosum* (right), LM (a) and SEM (b). Scale bars = 10 μm.

**TABLE 2:** Morphological comparison of *Prorocentrum majusculum* n. sp. with other species in *P. cordatum* group.

	*P. majusculum*	*P. cordatum*	*P. insidiosum*	*P. pervagatum*	*P. shikokuense*	*P. spinulentum*	*P. thermophilum*
Cell outline in lateral view	Almost round	Ovate, rounded ovate	Round to ovate	Asymmetrically oval to round	Similar to a spatula without a handle	Irregularly oval to round	Almost round
Cell length (mean and min to max range) (μm)	23.0 (17.3–28.8)	(10–18)	13.8 (11.8–16.0)	10.6–13.9 (8.3–16.0)	(15.3–19.9)	10.9 (9.0–12.8)	13.9 (11.8–16.6)
Cell depth (mean and min to max range) (μm)	22.7 (16.9–28.8)	(10–18)	12.0 (8.0–15.2)	10.2–14.6 (8.0–15.9)	(8.1–9.4)	10.3 (8.5–11.9)	12.3 (9.8–15.2)
Nucleus outline and position	Round/oval, posterior	Round/oval, posterior	Round/oval, posterior	Round/oval, posterior	Round, posterior	Oval, median/sub‐median	Round/oval, posterior
Pyrenoid number and position	Two, both sides	n.a.	Two	Two	n.a.	No	n.a.
Protrusion of platelet 8	ap‐ and fp‐facing sides	ap‐ and fp‐facing sides	ap‐facing side only	ap‐ and fp‐facing sides	No protrusion	ap‐ and fp‐facing sides	ap‐ and fp‐facing sides
Highest protrusion on platelet	1	1	1, 6	6	1	1	1
Protrusion on platelet 1	Two, similar in size	Two, similar in size	Two, inner one longer	Two, inner one longer	One	One	Two, similar in size
References	This study	Pertola et al. (2003), Monti et al. (2010), Larsson et al. (2025)	Larsson et al. (2025)	Tillmann, Wietkamp, et al. (2023)	Roselli et al. (2019), Shin et al. (2019)	Tillmann, Gottschling, et al. (2023)	Gómez et al. (2023), Larsson et al. (2025)

Abbreviations: ap, accessory pore; fp, flagellar pore.

All members within the *P. cordatum* group, including *P. majusculum*, possess eight platelets in the periflagellar area (with the possible exception of *P. shikokuense*, where a subdivision of platelet 5 has been suggested (Shin et al. 2019)), and most of them have a characteristic double‐wing structure on platelet 1 (Larsson et al. 2025; Figure 10). While this pattern is highly conserved, subtle but consistent differences in shape in platelet projections provide useful characters for species delimitation. In *P. majusculum*, the apical protrusion (i.e., a projection that is higher than it is wide) on platelet 6 terminates in a flat top, differing from the pointed, spine‐like protrusion in *P. cordatum* (Pei et al. 2022) and the conspicuously long apical spine in *P. pervagatum* (Tillmann, Wietkamp, et al. 2023). In *P. spinulentum*, the outer wing on platelet 1 is much higher than the inner wing, which forms only low lists outlining the ap (Tillmann, Gottschling, et al. 2023; Larsson et al. 2025), whereas both wings are of similar height in *P. majusculum*. Comparing *P. majusculum* with *P. insidiosum*, there are two differences in platelet projections: *P. majusculum* has two wings on platelet 8 lining both the accessory and flagellar pores, rather than a single wing along the ap side only for *P. insidiosum* (Larsson et al. 2025). Moreover, *P. majusculum* has a shorter inner wing on platelet 1 than that of *P. insidiosum*. The periflagellar projections in *P. thermophilum* differ from *P. majusculum* (and other *P. cordatum* group members) in that they are much smaller, less prominent, and more complex, forming several flat wings on platelet 1 (Gómez et al. 2023; Larsson et al. 2025).

**FIGURE 10:**
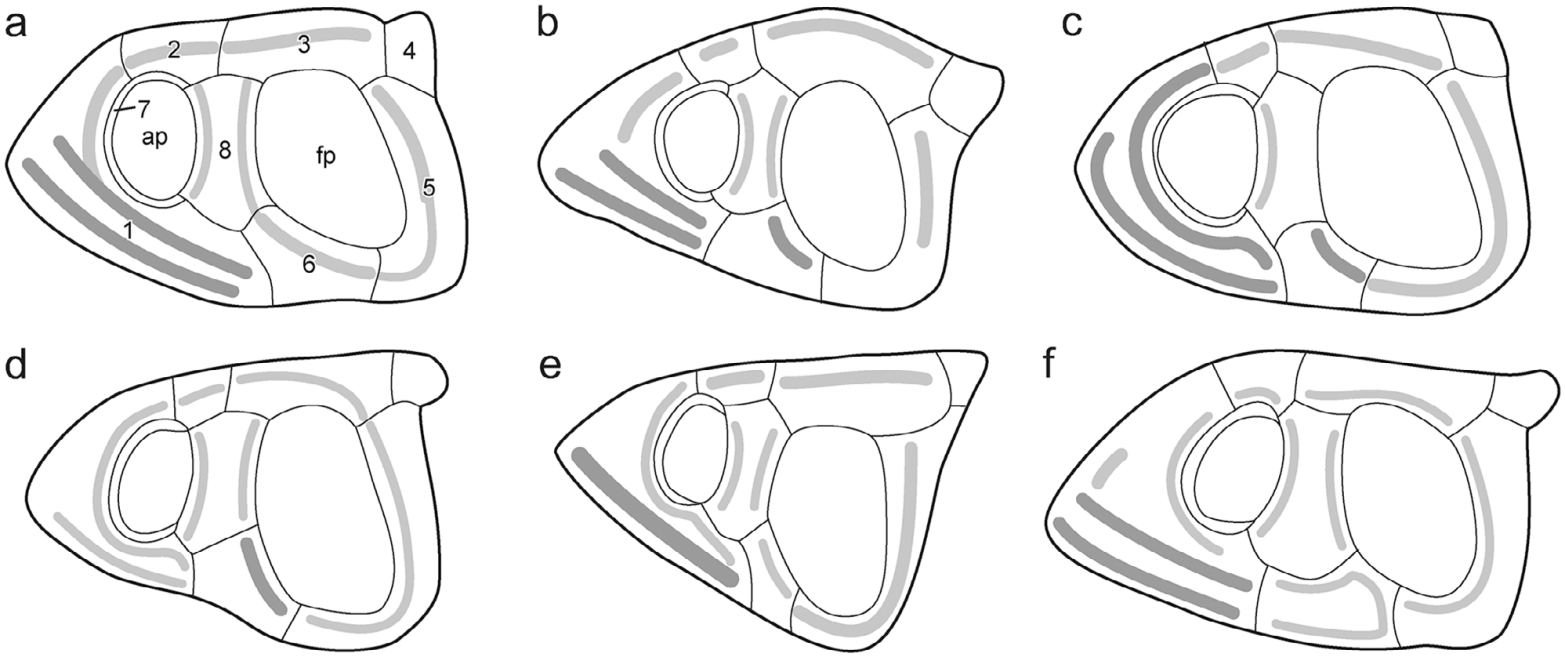
Schematic illustrations of the periflagellar area of *Prorocentrum majusculum* n. sp. and related round and spiny species. (a) *P. majusculum*. (b) *P. cordatum* . (c) *P. insidiosum*. (d) *P. pervagatum*. (e) *P. spinulentum*. (f) *P. thermophilum* . Dark and light gray lines indicate taller and shorter wing‐like protrusions, respectively. The arrangements of related species are given according to previous studies (Monti et al. 2010; Tillmann, Gottschling, et al. 2023; Tillmann, Wietkamp, et al. 2023; Larsson et al. 2025).

While the morphological differences of *P. majusculum* clearly separate it from other species in the *P. cordatum* group, it is worth highlighting the traits that these species have in common. As discussed in detail by Larsson et al. (2025), species within the *P. cordatum* group are generally roundish in lateral view, although *P. shikokuense* exceptionally shows notable elongation (Hada 1975). *Prorocentrum majusculum* thus fits well within the typical shape range of the group but generally has a larger cell size than the other members, effectively expanding the known morphological spectrum of this lineage. All species within the *P. cordatum* group have similar ornamentation of the thecal plate surface with spines or knob‐like structures. The spines of *P. majusculum* closely resemble those of its closest phylogenetic relative, *P. insidiosum*, whereas the next closest species, *P. thermophilum* , exhibits more knob‐like ornamentation (Gómez et al. 2023; Larsson et al. 2025), indicating that spine versus knob morphology does not strictly reflect phylogenetic relationships.

### 4.2 Large and Small Thecal Pores

All species of the *P. cordatum* group possess two distinct types of thecal pores—small and large—that are readily distinguishable in internal views (Larsson et al. 2025). A characteristic feature is a group or short row of two to five large pores in an apical position on the ventral side of the right thecal plate (Larsson et al. 2025). *Prorocentrum majusculum* shares this feature, although the large pores rarely form a distinct row. As observed and discussed in *P. insidiosum* (Larsson et al. 2025), the large pores of *P. majusculum* often show only a small external opening. The size of the small pores is consistent across the group (ca. 0.12–0.16 μm; Gómez et al. 2023; Tillmann, Gottschling, et al. 2023; Larsson et al. 2025). The large pores of *P. majusculum* (0.40 μm in external view) are similar in size to those of its closest relative, *P. insidiosum* (0.41 μm; Larsson et al. 2025), and larger than those reported for *P. spinulentum* (0.23 μm; Tillmann, Gottschling, et al. 2023), *P. pervagatum* (0.26–0.34 μm; Tillmann, Wietkamp, et al. 2023), and *P. thermophilum* (ca. 0.30 μm; Gómez et al. 2023).

TEM observations of *P. majusculum* provided further insights into the structure and function of these two distinct thecal pore types. Vertical sections revealed that the marginal protrusion of external openings was apparently higher in the large pores (ca. 300 nm) compared to the small pores (ca. 50 nm). Moreover, after the secretory material had been discharged, the distal part of the external opening of the large pores remained narrower than the actual pore chamber. In contrast, the external opening of the small pores was similar in diameter to the pore chamber. This may explain why large pores appear smaller than their actual size in external SEM views of the theca (Larsson et al. 2025). Additionally, electron‐lucent material was observed at the internal opening of small pores, narrowing the discharging canal and partly obstructing the empty canal; this is consistent with the smaller or closed small pores observed in internal SEM views of thecal plates.

TEM observations of *P. majusculum* suggested that electron‐dense granules are associated with the small pores, whereas trichocysts with characteristic striated bands are associated with the large pores. Although the function of the small pores remains unclear, the electron‐dense granular material in the chambers beneath them resembles the contents of the small Type C mucocysts (Table 3). Regarding the large pores, flask‐shaped vesicles have been reported beneath the large pores in the benthic species *P. tsawwassenense* (Hoppenrath and Leander 2008). In contrast, TEM observations of the planktonic species *P. texanum* suggested that the large pores function as trichocyst pores (Henrichs et al. 2013), and SEM observations directly captured trichocyst discharge through the large pores in *P. micans* (Westermann et al. 2015). Together with the present observations, these findings support the interpretation that the large pores of planktonic *Prorocentrum* species function as trichocyst pores.

**TABLE 3:** Mucocyst types of dinoflagellates based on shape, position, and contents.

	Type A: Solitary, elongated mucocysts, peripherally distributed, with reticulated, granular, or fibrous contents	Type B: Clustered mucocysts, with fibrous contents. Some species associated with a tubular canal (*)	Type C: Solitary, small mucocysts peripherally positioned, usually flask‐shaped, with granular contents
Amphidiniales			*Amphidinium carterae* (Klut et al. 1985)
Gymnodiniales sensu lato	*Actiniscus pentasterias* (Hansen 1993), *Barrufeta bravensis* (Sampedro et al. 2011), *Bispinodinium angelaceum* (Yamada et al. 2013), *Gymnodinium dorsalisulcum* (Luo et al. 2018), *G. fuscum* (Hansen et al. 2000), *Gyrodinium spirale* (Hansen and Daugbjerg 2004), *Lepidodinium viride* (Watanabe et al. 1987, 1990), *L. chlorophorum* (Elbrächter and Schnepf 1996; Honsell and Talarico 2004), *Margalefidinium polykrikoides* (Iwataki et al. 2010), *Nematopsides vigilans* (Moestrup et al. 2026), *Nusuttodinium myriopyrenoides* (Yamaguchi et al. 2011), *Polykrikos lebourae* (Hoppenrath and Leander 2007), *Spiniferodinium palauense* (Horiguchi et al. 2011)		*Actiniscus pentasterias* (Hansen 1993), *Apicoporus parvidiaboli* (Sparmann et al. 2008), *Barrufeta resplendens* (Gu et al. 2015), *Lepidodinium chlorophorum* (Elbrächter and Schnepf 1996)
Suessiales	*Baldinia anauniensis* (Hansen et al. 2007)		*Ansanella natalensis* (Dawut et al. 2018; Horiguchi and Pienaar 1994), *Sphaerodinium cracoviense* (Craveiro et al. 2010)
Prorocentrales		*Prorocentrum concavum* (Mohammad‐Noor et al. 2007), *P. cordatum* (Berdieva et al. 2018; Dodge and Bibby 1973), *P. emarginatum* (Hoppenrath et al. 2013), *P. lima* (Hoppenrath and Leander 2008; Hoppenrath et al. 2013), *P. majusculum* (this study), *P. micans* * (Bouck and Sweeney 1966; Dodge and Bibby 1973), *P. nux* (Puigserver and Zingone 2002), *P. rhathymum* (Hoppenrath et al. 2013), *P. texanum* var. *texanum* (Henrichs et al. 2013), *P. tsawwassenense* (Hoppenrath and Leander 2008)	*Prorocentrum lima* , *P. maculosum* (Zhou and Fritz 1993), *P. tsawwassenense* (Hoppenrath and Leander 2008)
Peridiniales sensu lato		Azadinium inconspicuum*, *A. spinosum* *, A. poporum* (Kuwata et al. 2026), *Oxytoxum lohmannii* (Cooney et al. 2024)	*Palatinus apiculatus* (Craveiro et al. 2009)
Gonyaulacales		*Gambierdiscus toxicus* *, *Coolia monotis* * (Besada et al. 1982), *Ostreopsis* cf. ovata* (Besada et al. 1982; Escalera et al. 2014; Honsell et al. 2013)	*Alexandrium catenella* (Hansen and Moestrup 1998), *Ostreopsis* cf. *ovata* (Honsell et al. 2013)

Numerous trichocysts of various sizes were observed in *P. majusculum*; trichocysts generally represent a characteristic feature of *Prorocentrum* species. In addition, vesicles containing square‐profile crystalline lattice structures, presumed to represent trichocyst precursors, were identified. Similar vesicles have previously been reported in *Durinskia dybowskii* (as *Peridinium balticum* ), *Lingulaulax polyedra* (as *Gonyaulax polyedra* ), and *Hematodinium* sp. (Bouck and Sweeney 1966; Tomas and Cox 1973; Gaudet et al. 2014). These vesicles have been interpreted as successive developmental stages of trichocyst formation, progressing from primordial vesicles containing granular material to mature trichocysts (Tomas and Cox 1973; Gaudet et al. 2014). Further ultrastructural studies are needed to clarify the developmental process of trichocysts in *Prorocentrum*.

### 4.3 Mucus Traps

Mixotrophic feeding via mucus trap formation has been documented in several members of the *P. cordatum* group, including *P. insidiosum*, *P. cordatum* , and *P. pervagatum* (Larsson et al. 2022; Tillmann, Mitra, et al. 2023). Observations by Funaki et al. (2024) of mucus trap formation in a strain of *P. majusculum* (as *P*. cf. *balticum*) extend this feeding strategy to an additional species. In their study, mucus trap formation was observed during co‐culture with the cryptophyte *Rhodomonas*; however, direct evidence of phagotrophic mixotrophy, such as peduncle formation or prey ingestion, has not been demonstrated for *P. majusculum*. In the present study, mucocysts and peduncle‐associated structures were observed in *P. majusculum*, providing additional ultrastructural evidence for a potential role in phagotrophic feeding.

### 4.4 Mucocysts

Mucocysts have been observed in various dinoflagellate groups and have been described using different terms, including vesicles containing a fibrous matrix (e.g., Puigserver and Zingone 2002). Another comparable structure, the fibrous vesicle (also termed fibrous body), has been reported from various dinoflagellates and is distinguishable from mucocysts by its elongated shape and the parallel arrangement of its internal fibers along its longitudinal axis (e.g., Dodge 1971; Kuwata, Hernández‐Becerril, et al. 2024; Kuwata, Lum, et al. 2024; Kuwata et al. 2026; Grzebyk et al. 2025; Takahashi et al. 2025). Mucocyst‐like structures in dinoflagellates exhibit significant morphological variation, differing in size, shape, and internal contents. To the best of our knowledge, however, distinct mucocyst morphologies appear to be characteristic of different dinoflagellate groups (Table 3). Here we distinguish three types (Types A–C) based primarily on their intracellular distribution and ultrastructural characteristics. In unarmored dinoflagellates such as *Gymnodinium*, *Gyrodinium*, and *Lepidodinium*, elongated mucocysts containing reticulated, granular, or fibrous material occur near the cell periphery (Type A). In contrast, armored dinoflagellates such as *Coolia*, *Gambierdiscus*, and *Prorocentrum* possess clustered mucocysts containing radially arranged fibrous material (Type B). Moreover, Type B mucocysts in *Azadinium*, *Coolia*, *Gambierdiscus*, *Ostreopsis*, and *P. micans* additionally contain a tubular canal (Dodge and Bibby 1973; Besada et al. 1982; Honsell et al. 2013; Escalera et al. 2014; Kuwata et al. 2026). The apparent difference between peripheral, non‐clustered mucocysts of unarmored species and the clustered mucocysts in armored species is intriguing. This distribution may indicate that the thecal pore alone is not sufficient for mucus secretion in thecate cells and that an additional structure, such as a tubular mucus canal, may be involved in mucus secretion. In *P. majusculum*, clustered mucocysts containing fibrous material (Type B) were observed around the flagellar region, but their connection to the flagellar or accessory pores was not clearly discernible. A third type (Type C) consists of small vesicles located beneath the amphiesma. Similar vesicles have been reported in various dinoflagellate groups. They typically contain granular material and resemble the vesicles associated with the small thecal pore of *P. majusculum*.

In *Prorocentrum*, Type B and C mucocysts have been reported (Table 3). These correspond to Type 1 and 2, respectively, described in *P. tsawwassenense* (Hoppenrath and Leander 2008). Small Type C mucocysts have been reported from several benthic *Prorocentrum* species, including *P. lima* , *P. maculosum* , and *P. tsawwassenense* (Zhou and Fritz 1993; Hoppenrath and Leander 2008). Type B mucocysts similar to those observed in *P. majusculum* have been reported from *P. cordatum* (as *Exuviaella mariae‐lebouriae* in Dodge and Bibby 1973; as *P. minimum* in Berdieva et al. 2018). Additionally, they have been found in other planktonic species including *P. micans* , *P. nux* , and *P. texanum* (Bouck and Sweeney 1966; Dodge and Bibby 1973; Puigserver and Zingone 2002; Henrichs et al. 2013), as well as in benthic species such as *P. concavum* , *P. lima* , *P. rhathymum*, and *P. tsawwassenense* (Mohammad‐Noor et al. 2007; Hoppenrath and Leander 2008; Hoppenrath et al. 2013; Table 3). Among these *Prorocentrum* species, the size and fibrous contents of Type B mucocysts in *P. cordatum* most closely resemble those in *P. majusculum* (Dodge and Bibby 1973; Berdieva et al. 2018). As both species are known to form mucus traps (Tillmann, Mitra, et al. 2023; Funaki et al. 2024), Type B mucocysts may be the source of the mucus forming the traps.

### 4.5 Peduncle

A peduncle is a tubular cellular extension involved in feeding, such as myzocytosis and micropredation, and has been reported in various dinoflagellate genera, e.g., *Azadinium*, *Dactylodinium*, *Dinophysis*, *Gymnodinium*, *Karlodinium*, *Nematopsides*, *Oxyrrhis*, *Oxytoxum*, and *Peridiniopsis* (Brown et al. 1988; Hansen 2001; Calado and Moestrup 2002; Garcia‐Cuetos et al. 2010; Takahashi et al. 2017; Lum et al. 2019; Benico et al. 2020; Cooney et al. 2024; Kuwata et al. 2026; Moestrup et al. 2026). In *P. majusculum*, the microtubular strand of peduncle (MSP) and associated electron‐opaque and electron‐lucent vesicles were observed. The structure has been reported from several *Prorocentrum* species, such as *P. cordatum* (as *E. mariae‐lebouriae* ) and *P. micans* (Dodge and Bibby 1973; Roberts et al. 1995), suggesting that peduncular feeding may be common within the genus. Direct evidence for peduncular feeding, that is, the content of prey cells being sucked into the *Prorocentrum* cell, was observed for other mucus trap‐forming species, including *P. insidiosum* (Larsson et al. 2022) as well as *P. pervagatum* and *P. cordatum* (Tillmann, Mitra, et al. 2023). In contrast, direct engulfment was described in *P. micans* and *P. shikokuense* (as *P. donghaiense*) by Jeong et al. (2005).

In *Prorocentrum*, the peduncle extrudes from the apical area (Tillmann, Mitra, et al. 2023), but the emerging side, whether from the flagellar canal or the accessory canal, has remained unclear (Roberts et al. 1995; Hoppenrath et al. 2013). The present TEM observation showed that the distal end of the MSP, surrounded by the peduncle striated collar (PSC), terminated in the flagellar canal below the lip‐like structure. The MSP, a bundle of microtubules, can be distinguished from other microtubule strands, such as the longitudinal microtubular root, by its association with electron‐dense material. Electron‐dense material has been observed in proximity to the MSP of various dinoflagellates, for example, *Azadinium*, *Dactylodinium*, *Karlodinium*, *Pfiesteria*, and *Prorocentrum* (Dodge and Bibby 1973; Vogelbein et al. 2002; Berge et al. 2012; Lum et al. 2019; Benico et al. 2020; Kuwata et al. 2026). Semi‐serial sections further showed that the MSP of *P. majusculum* extends from the ventral side below the wing‐like protrusion of platelet 1, although the proximal termination could not be traced.

## Author Contributions

Koyo Kuwata: writing – review and editing, writing – original draft, resources, investigation, funding acquisition, formal analysis, data curation. Urban Tillmann: writing – review and editing, writing – original draft, investigation. Chiho Funaki: writing – review and editing, investigation. Takako Masuda: writing – review and editing, resources. Kazutaka Takahashi: writing – review and editing. Mitsunori Iwataki: writing – review and editing, writing – original draft, supervision, project administration, methodology, investigation, funding acquisition, conceptualization.

## Funding

This work was supported by Japan Society for the Promotion of Science (JSPS) KAKENHI 24KJ0853 (KK), 23KK0117, 26H02333 (MI), and 24H00075 (KT).

## Supporting information


**Figure S1:** Measurement points of thecal pore size (yellow: small pore, red: large pore). (a) External view. (b) Internal view. Scale bars = 2 μm.
**Figure S2:** Light microscopy of *Prorocentrum majusculum* n. sp. strain KWT168. (a–c) Deeper foci showing pyrenoid (Py), nucleus (Nu), and trichocysts (black arrowheads). (d) Apical view showing accessory pore (white arrow) and flagellar pore (black arrow). (e) A cell and extruded trichocysts (black arrowheads). Scale bars = 5 μm.
**Figure S3:** Transmission electron microscopy of *Prorocentrum majusculum* n. sp., strain KWT168. (a, b) Vesicles containing the trichocyst precursor. (c, d) Mucocysts containing electron‐lucent and electron‐dense materials. Scale bars = 500 nm (a, d), 2 μm (b), 1 μm (c).
**Figure S4:** Bayesian inference tree of *Prorocentrum* species based on SSU rDNA sequence (1817 bp). Posterior probabilities (> 0.70) of Bayesian inference (BI), and ultrafast bootstrap support values (≥ 90%) of ML are given at nodes (BI/ML), and black dots indicate maximum supports (BI/ML = 1.00/100). DNA sequences determined in the present study are highlighted in black. *NA, locality information not available.
**Figure S5:** Bayesian inference tree of *Prorocentrum* species based on ITS region sequence (606 bp). Posterior probabilities (> 0.70) of Bayesian inference (BI), and ultrafast bootstrap support values (≥ 90%) of ML are given at nodes (BI/ML), and black dots indicate maximum supports (BI/ML = 1.00/100). DNA sequences determined in the present study are highlighted in black. *NA, locality information not available.
**Figure S6:** Bayesian inference tree of *Prorocentrum* species based on LSU rDNA sequence (1257 bp). Posterior probabilities (> 0.70) of Bayesian inference (BI), and ultrafast bootstrap support values (≥ 90%) of ML are given at nodes (BI/ML), and black dots indicate maximum supports (BI/ML = 1.00/100). DNA sequences determined in the present study are highlighted in black. *NA, locality information not available.
**Figure S7:** ITS2 secondary structure model of (a) *Prorocentrum majusculum* n. sp. strain KWT167 and (b) *P. insidiosum* strain UTSPH3C1 showing four helices (I, II, III, and IV). The location of hemi‐compensatory base changes (hCBC) is indicated by arrows.
**Table S1:** Genetic distance (*p*‐distance) inferred from the ITS region (554 bp) of the *Prorocentrum cordatum* group.

## Data Availability

The data that support the findings of this study are available in the Supporting Information of this article.
