# Supplementary material for: Thecal and Intracellular Ultrastructure of Prorocentrum majusculum n. sp. (Prorocentrales, Dinophyceae), a New Spiny Prorocentrum from Northwest Pacific
Source: J Eukaryot Microbiol. 2026 Nov 14;73(6):e70141. doi: 10.1111/jeu.70141 (PMC13768824; doi:10.1111/jeu.70141)
Supplement: Supplementary file 1 [file JEU-73-e70141-s001.pdf]

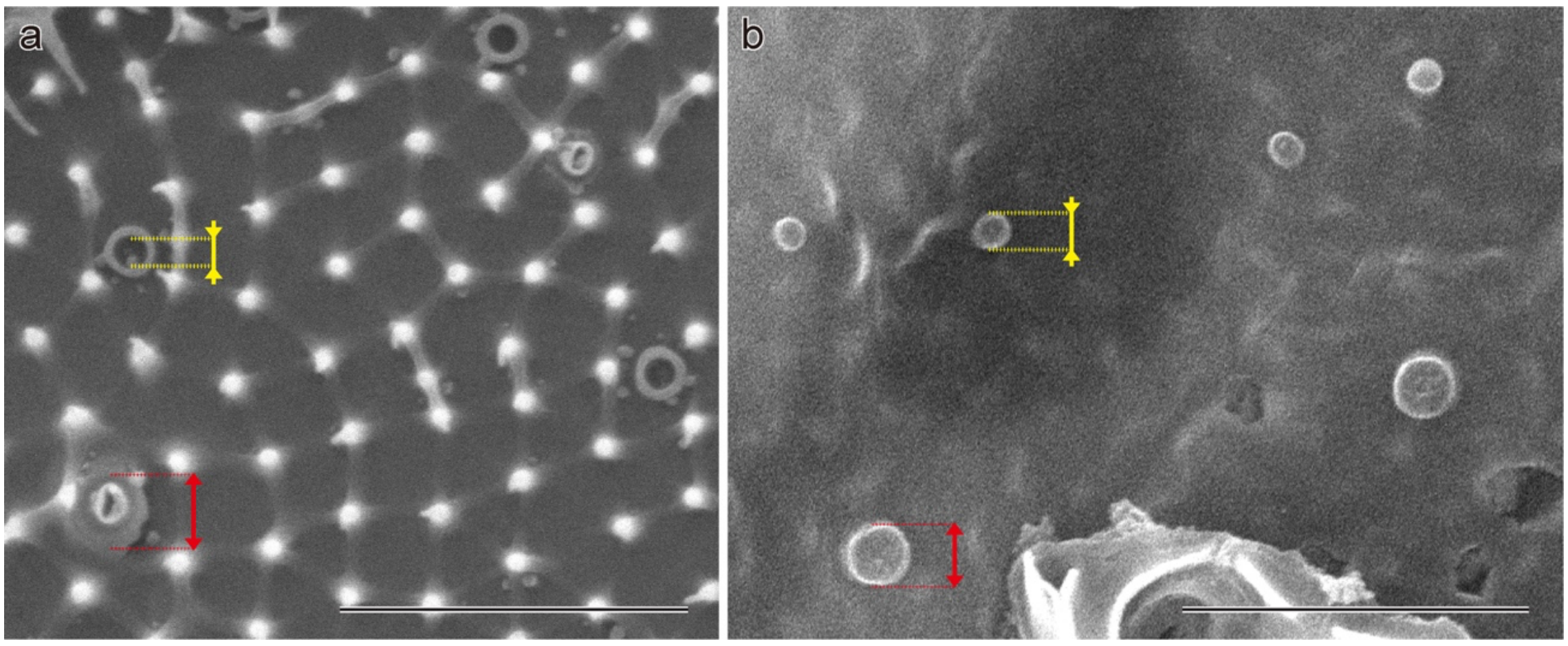

FIGURE S1 Measurement points of thecal pore size (yellow: small pore, red: large pore). (a) External view. (b) Internal view. Scale bars = 2 μm.

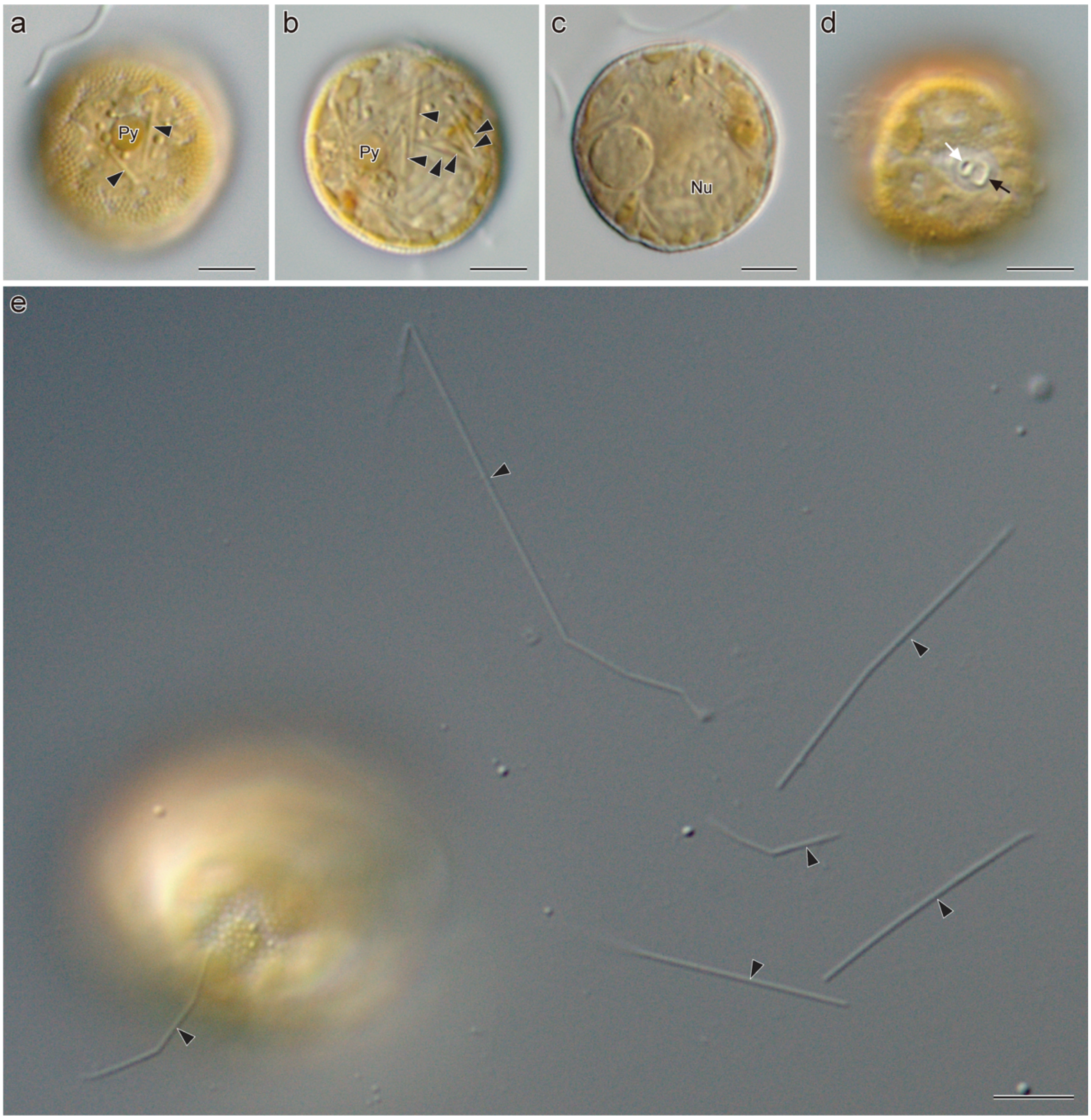

FIGURE S2 Light microscopy of *Prorocentrum majusculum* n. sp. strain KWT168. (a–c) Deeper foci showing pyrenoid (Py), nucleus (Nu), and trichocysts (black arrowheads). (d) Apical view showing accessory pore (white arrow) and flagellar pore (black arrow). (e) A cell and extruded trichocysts (black arrowheads). Scale bars = 5 μm.

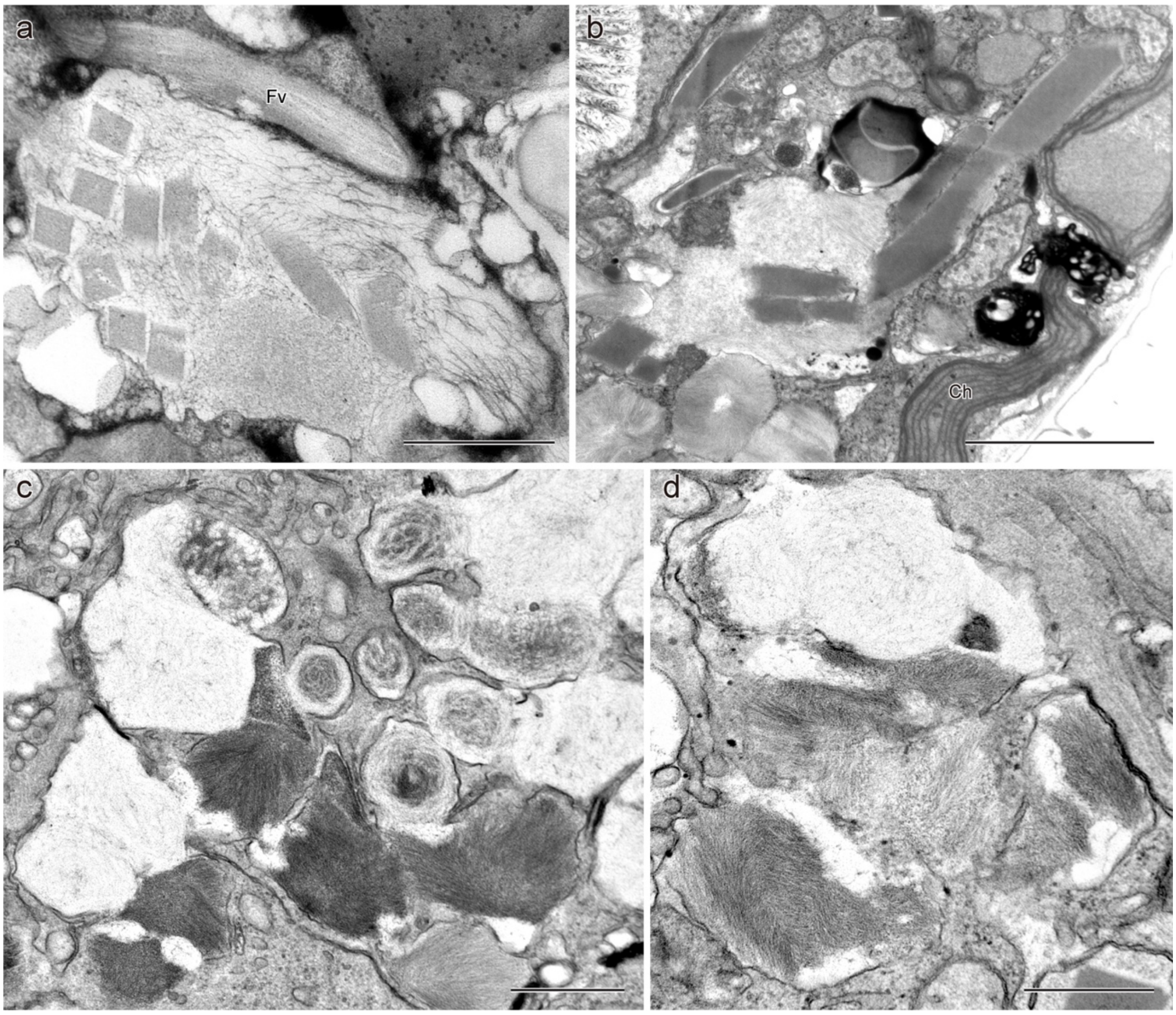

FIGURE S3 Transmission electron microscopy of *Prorocentrum majusculum* n. sp., strain KWT168. (a, b) Vesicles containing the trichocyst precursor. Fibrous vesicle (Fv) is indicated. (c, d) Mucocysts containing electron-lucent and electron-dense materials. Scale bars = 500 nm (a, d), 2 µm (b), 1 µm (c).

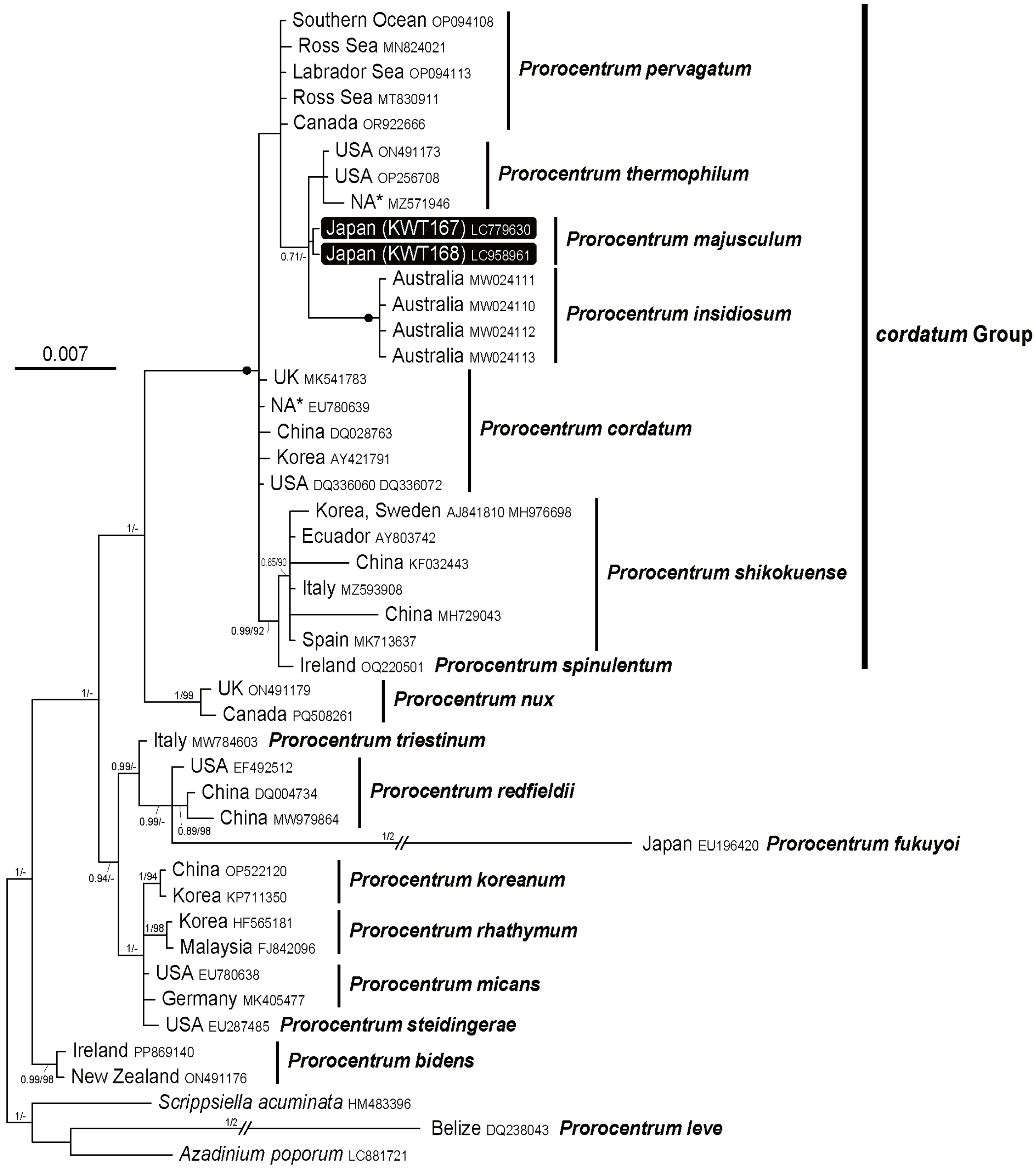

FIGURE S4 Bayesian inference tree of *Prorocentrum* species based on SSU rDNA sequence (1817 bp). Posterior probabilities (>0.70) of Bayesian inference (BI), and ultrafast bootstrap support values (≥90 %) of ML are given at nodes (BI/ML), and black dots indicate maximum supports (BI/ML = 1.00/100). DNA sequences determined in the present study are highlighted in black. *NA: locality information not available.

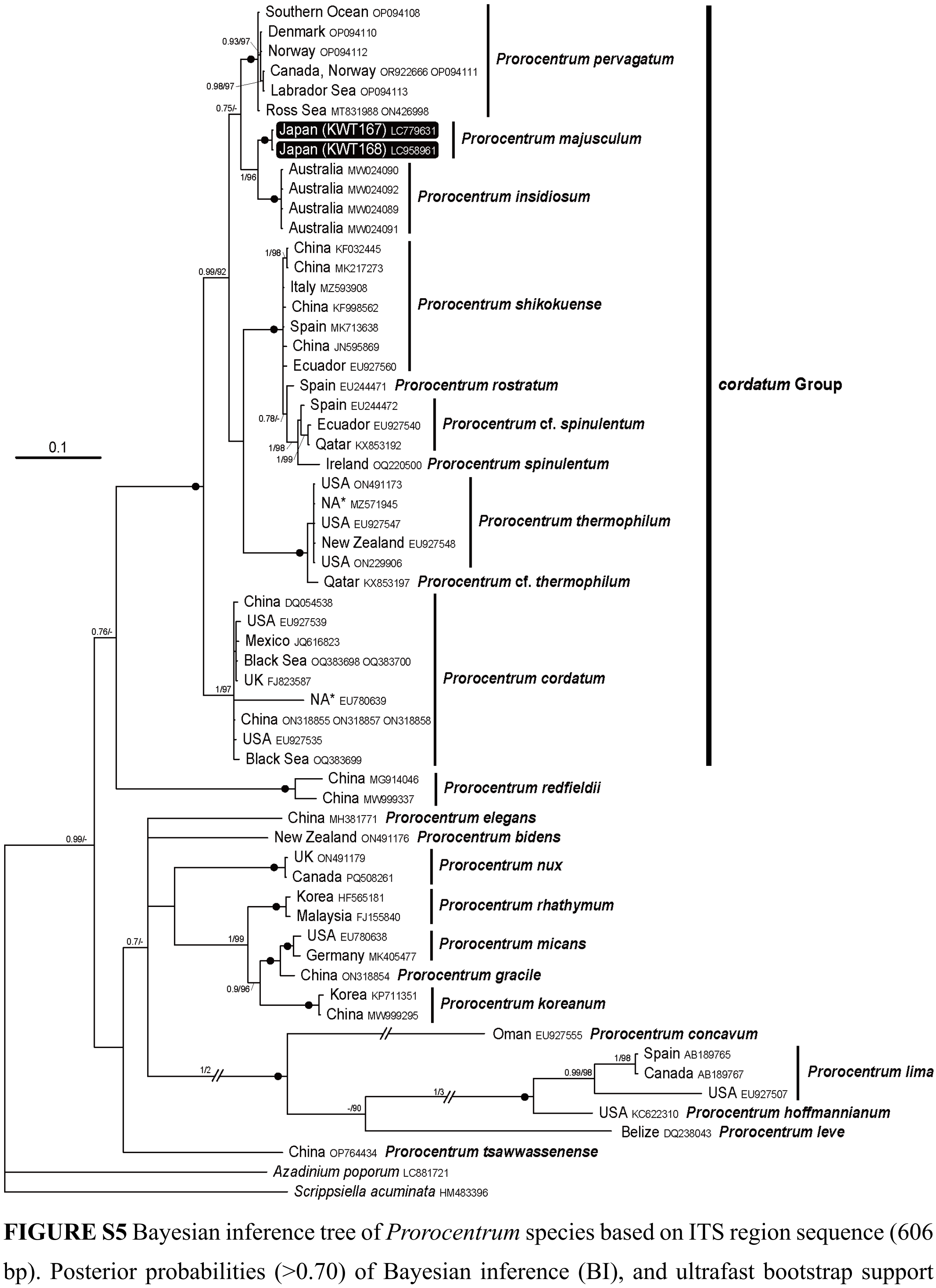

FIGURE S5 Bayesian inference tree of *Prorocentrum* species based on ITS region sequence (606 bp). Posterior probabilities (>0.70) of Bayesian inference (BI), and ultrafast bootstrap support values (≥90 %) of ML are given at nodes (BI/ML), and black dots indicate maximum supports (BI/ML = 1.00/100). DNA sequences determined in the present study are highlighted in black. *NA: locality information not available.

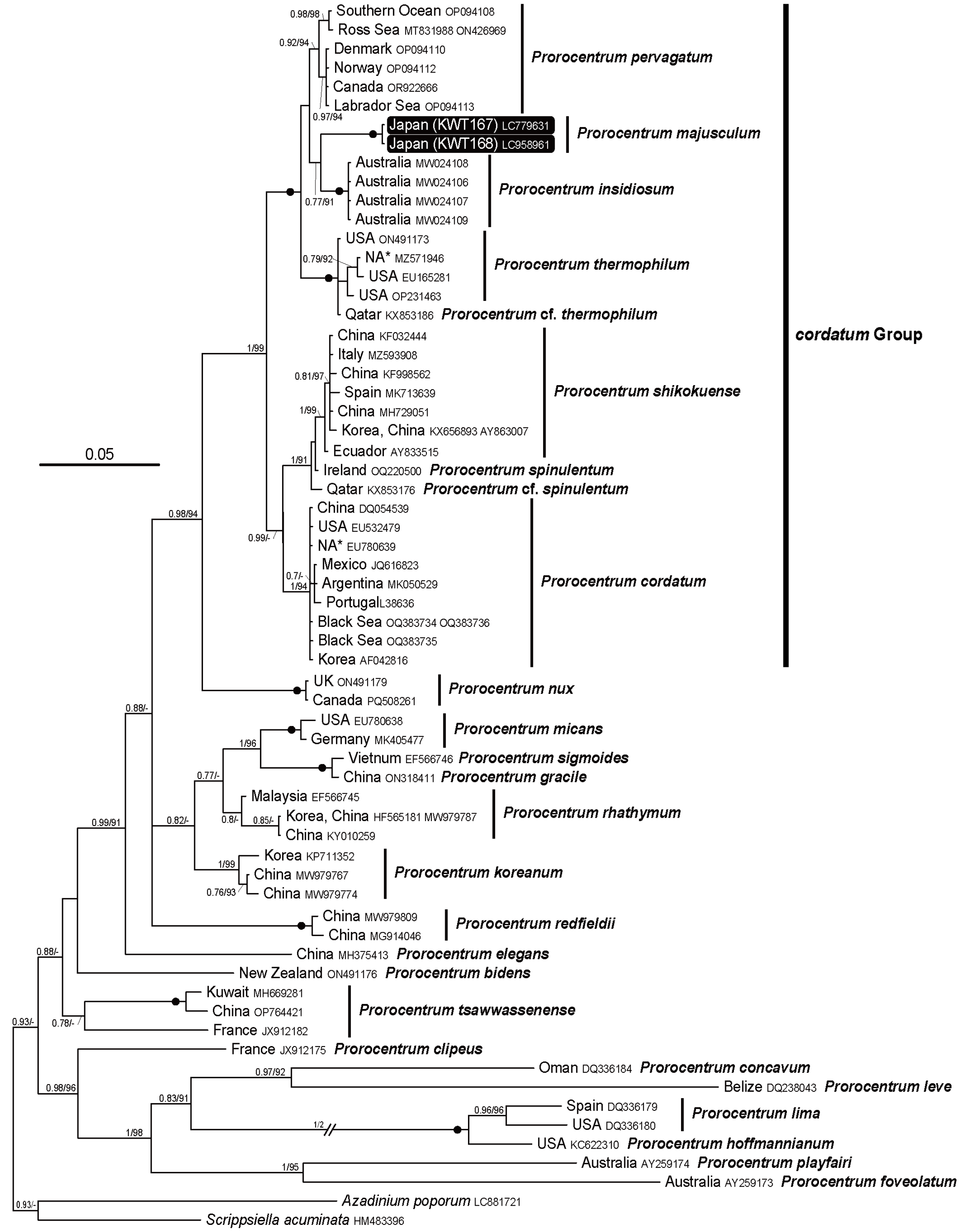

FIGURE S6 Bayesian inference tree of *Prorocentrum* species based on LSU rDNA sequence (1257 bp). Posterior probabilities (>0.70) of Bayesian inference (BI), and ultrafast bootstrap support values (≥90 %) of ML are given at nodes (BI/ML), and black dots indicate maximum supports (BI/ML = 1.00/100). DNA sequences determined in the present study are highlighted in black. *NA: locality information not available.

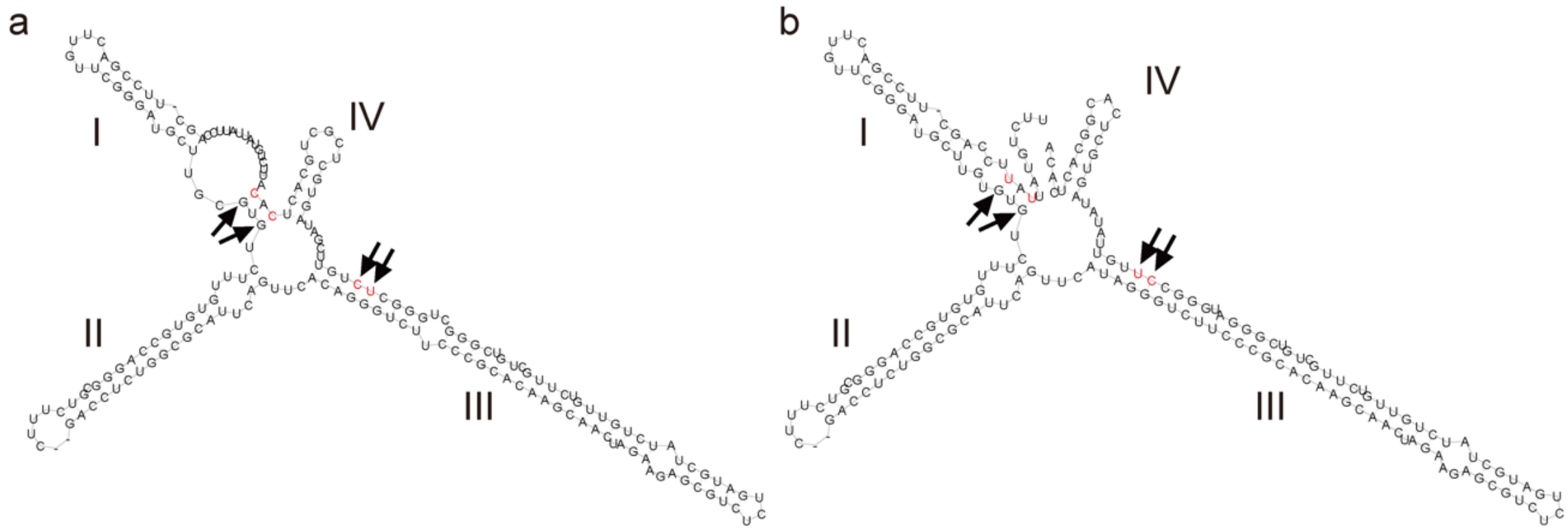

FIGURE S7 ITS2 secondary structure model of (a) *Prorocentrum majusculum* n. sp. strain KWT167 and (b) *P. insidiosum* strain UTSPH3C1 showing four helices (I, II, III, and IV). The location of hemi-compensatory base changes (hCBC) is indicated by arrows.

TABLE S1 Genetic distance (*p*-distance) inferred from the ITS region (554 bp) of the *Prorocentrum cordatum* group.

	P. cordatum[a]	*P. insidiosum*[b]	*P. pervagatum*[c]	*P. shikokuense*[d]	*P. spinulentum*[e]	*P. thermophilum*[f]
P. insidiosum	0.099–0.104					
P. pervagatum	0.082–0.089	0.062–0.065				
P. shikokuense	0.086–0.097	0.090–0.093	0.075–0.084			
P. spinulentum	0.121–0.126	0.119	0.108–0.113	0.042–0.046		
P. thermophilum	0.121–0.128	0.100–0.102	0.102–0.107	0.104–0.110	0.133–0.135	
P. majusculum[g]	0.097–0.102	0.040	0.049–0.053	0.095–0.097	0.126	0.091–0.093

Note: strain name, GenBank accession number, and reference used in this analysis.

[a]PMDH01 (DQ054538; Hou et al. unpubl.), PIPV1 (JQ616823; Herrera-Sepúlveda et al. 2013), CCMP1329 (EU927539; Ferrell and Beaton unpubl.), TIO348 (ON318858; Pei et al. unpubl.), TIO354 (ON318855; Pei et al. unpubl.), CCMP696 (EU927535; Ferrell and Beaton unpubl.), BS4-A5 (OQ383698; Tillmann et al. 2023b), CCAP1136/16 (EU927545; Ferrell and Beaton unpubl.)

[b]UTSPH3C1 (MW024090; Larsson et al. 2022)

[c]CA01 (OP094108; Tillmann et al. 2023c), LPD10 (OP094110; Tillmann et al. 2023c), P3B7 (OP094111; Tillmann et al. 2023c), DINO:1 (MT831988; Bolinesi et al. 2020; Gómez et al. 2023)

[d]MABT-1 (KF032445; Chen et al. 2013), Prosh-18S-28S_June2 (MZ593908; Zhang et al. unpubl.), KF998562 (no strain information; Yuan et al. 2015), YJ2 (JN595869; Yuan et al. 2012), CCMP1517 (EU927560; Ferrell and Beaton unpubl.)

[e]1D3 (OQ220500; Tillmann et al. 2023a)

[f]CCMP1260 (EU927547; Ferrell and Beaton unpubl.), CCMP1787 (EU927548; Ferrell and Beaton unpubl.)

[g]KWT167 (LC779631; Funaki et al. 2024; this study)

Reference

Bolinesi F., M. Saggiomo, S. Aceto, A. Cordone, E. Serino, M. C. Valoroso, and O. Mangoni. 2020. “On the Relationship between a Novel *Prorocentrum* sp. and Colonial *Phaeocystis antarctica* under Iron and Vitamin B12 Limitation: Ecological Implications for Antarctic Waters.” *Applied Sciences* 10: 6965.

Chen G., C. Ma, C. Zhang, J. Zhou, Y. Wang, G. Wang, B. Zhang, Z. Xu, and D. D. Lu. 2013. “A Rapid and Sensitive Method for Field Detection of *Prorocentrum donghaiense* Using Reverse Transcription-Coupled Loop-Mediated Isothermal Amplification.” *Harmful Algae* 29: 31–39.

Funaki C., K. Kuwata, W. M. Lum, K. Takahashi, T. Masuda, M. Ichinomiya, T. Katayama, T. Kodama, M. Iwataki, and K. Takahashi. 2024. “Effects of Temperature and Light Intensity on Growth in *Prorocentrum* cf. *balticum* (Dinophyceae) Isolated from the Oyashio-Kuroshio Mixed Water Region, North Pacific.” *Plankton and Benthos Research* 19: 191–202.

Gómez F., P. Gourvil, T. Li, Y. Huang, H. Zhang, L. Courcot, L. F. Artigas, E. Soler Onís, A. Gutiérrez-Rodríguez, and S. Lin. 2023. “Molecular Phylogeny of the Spiny-Surfaced Species of the Dinoflagellate *Prorocentrum* with the Description of *P. thermophilum* sp. nov. and *P. criophilum* sp. nov. (Prorocentrales, Dinophyceae).” *Journal of Phycology* 59: 70–86.

Herrera-Sepúlveda A., N. Y. Hernandez-Saavedra, L. K. Medlin, and N. West. 2013. “Capillary Electrophoresis Finger Print Technique (CE-SSCP): an Alternative Tool for the Monitoring Activities of HAB Species in Baja California Sur Costal.” *Environmental Science and Pollution Research* 20: 6863–6871.

Larsson M. E., A. R. Bramucci, S. Collins, G. Hallegraeff, T. Kahlke, J. B. Raina, J. R. Seymour, and M. A. Doblin. 2022. “Mucospheres Produced by a Mixotrophic Protist Impact Ocean Carbon Cycling.” *Nature Communications* 13: 1–15.

Tillmann U., M. Gottschling, S. Wietkamp, and M. Hoppenrath. 2023. “Morphological and Phylogenetic Characterisation of *Prorocentrum spinulentum*, sp. nov. (Prorocentrales, Dinophyceae), a Small Spiny Species from the North Atlantic.” *Microorganisms* 11: 271.

Tillmann U., A. Mitra, K. J. Flynn, and M. E. Larsson. 2023. “Mucus-Trap-Assisted Feeding is a Common Strategy of the Small Mixoplanktonic *Prorocentrum pervagatum* and *P. cordatum* (Prorocentrales, Dinophyceae).” *Microorganisms* 11: 1–24.

Tillmann U., S. Wietkamp, M. Gottschling, and M. Hoppenrath. 2023. “*Prorocentrum pervagatum* sp. nov. (Prorocentrales, Dinophyceae): A New, Small, Planktonic Species with a Global Distribution.” *Phycological Research* 71: 56–71.

Yuan J., M. Li, and S. Lin. 2015. “An Improved DNA Extraction Method for Efficient and

Quantitative Recovery of Phytoplankton Diversity in Natural Assemblages." *PLOS ONE* 10: 1–18.

Yuan J., T. Mi, Y. Zhen, and Z. Yu. 2012. "Development of a Real-Time PCR Method (Taqman) for Rapid Identification and Quantification of *Prorocentrum donghaiense*." *Journal of Ocean University of China* 11: 366–374.
